# Associations between flavonoid-rich food and flavonoid intakes and incident unhealthy aging outcomes in older United States males and females

**DOI:** 10.1016/j.ajcnut.2025.02.010

**Published:** 2025-02-15

**Authors:** Nicola P Bondonno, Yan Lydia Liu, Francine Grodstein, Eric B Rimm, Aedín Cassidy

**Affiliations:** 1 Co-Centre for Sustainable Food Systems and Institute for Global Food Security, Queen’s University Belfast, North Ireland; 2 Institute for Nutrition Research, School of Medical and Health Sciences, Edith Cowan University, Perth, Australia; 3 Danish Cancer Society Research Centre (DCRC), Copenhagen, Denmark; 4 Department Nutrition, Harvard T.H. Chan School of Public Health, Boston, MA, United States; 5 Rush Alzheimer’s Disease Center, Rush University Medical Center, Chicago, IL, United States; 6 Department of Internal Medicine, Rush Medical College, Chicago, IL, United States; 7 Channing Division of Network Medicine, Brigham and Women’s Hospital and Harvard Medical School, Boston, MA, United States; 8 Department of Epidemiology, Harvard T.H. Chan School of Public Health, Boston, MA, United States

**Keywords:** flavonoids, flavodiet score, frailty, healthy aging, mental health, physical function

## Abstract

**Background:**

Our knowledge of the importance of flavonoid-rich foods in preventing unhealthy aging across its different domains is limited.

**Objectives:**

This study aimed to examine prospective associations between flavonoid-rich food and flavonoid intakes and indicators of unhealthy aging, namely frailty, impaired physical function, and poor mental health.

**Methods:**

We followed up 62,743 females and 23,687 males, all aged ≥60 y, from the Nurses’ Health Study (1990–2014) and Health Professionals Follow-up Study (2006–2018), respectively. Both time-updated and change in intakes of a flavodiet score (an aggregate of intakes of major flavonoid-rich foods and beverages) and individual flavonoid-rich foods and beverages and time-updated intakes of total flavonoids and flavonoid subclasses were calculated from food frequency questionnaires collected at baseline and every subsequent 4 y. Associations with incident frailty, impaired physical function, and poor mental health, assessed from self-reported questionnaire responses, were examined with multivariable-adjusted Cox proportional hazards models.

**Results:**

In the Nurses’ Health Study, participants with the highest flavodiet scores, compared with the lowest, had a 15% lower risk of frailty (HR_Q5vsQ1_: 0.85; 95%CI: 0.80, 0.90), a 12% lower risk of impaired physical function (HR_Q5vsQ1_: 0.88; 95% CI: 0.84, 0.91), and a 12% lower risk of poor mental health (HR_Q5vsQ1_: 0.88; 95% CI: 0.82, 0.94). Increases in flavodiet scores and both higher intakes and increases in intakes of tea, red wine, apples, blueberries, and oranges tended to be associated with lower risks of all outcomes. Higher intakes of total flavonoids and all flavonoid subclasses tended to be associated with a lower risk of each outcome. Although fewer associations were observed among males in the Health Professionals Follow-up Study, those with the highest flavodiet scores had a lower risk of poor mental health.

**Conclusions:**

High intakes of flavonoid-rich foods may support healthy aging. Further research is needed, including examining sex-specific associations, as incorporating flavonoid-rich foods in the diet may be a simple strategy to support healthy aging.

## Introduction

Amid prolonged life expectancy and declining fertility rates, a global shift toward older ages, termed population aging by the WHO, is underway [1]. On a biological level, aging results from the accumulation of molecular and cellular damage, leading to a gradual decline in physical and mental capacities, increased susceptibility to diseases, and eventual mortality [1]. Despite the increase in life expectancy, the proportion of these added years spent in good health has remained constant [2]. Therefore, recognizing the determinants of healthy aging is crucial for evidence-based prevention, aiming not just for extended lifespans but also prolonged healthspans.

Nutrition, particularly diets centered on plant-based foods, emerges as a critical determinant of health in aging [3,4]. Among the bioactive compounds present in plant-based foods, flavonoids have garnered attention for their potential impact on healthy aging [5,6]. Notably abundant in tea, apples, berries, citrus fruits, dark chocolate, and red wine, flavonoids demonstrate anti-inflammatory and oxidative stress reducing properties, suggesting that these foods may play a crucial role in mitigating age-related physiologic decline [5]. Additionally, flavonoids exhibit neuroprotective effects [7], offering potential protection against adverse mental health outcomes in older individuals. In the Nurses’ Health Study (NHS), higher midlife intakes of various flavonoid subclasses were associated with a higher likelihood of healthy aging, defined by survival to ≥70 y while maintaining 4 health domains (namely, the absence of major chronic diseases or impairments in cognitive, physical, or mental health) [8]. More recently, higher flavonoid intakes were associated with a deceleration in whole-body biological aging [9]. Despite the growing evidence supporting the health benefits of flavonoids, a comprehensive exploration of their association with specific domains of healthy aging, including frailty, impaired physical function, and poor mental health, remains an underexplored area. However, examining individual foods or compounds in isolation may overlook the cumulative or synergistic effects of flavonoid-rich dietary patterns. To address this, the flavodiet score was developed as a composite measure that captures overall adherence to a diet rich in flavonoid-containing foods [10], providing a more holistic perspective and facilitating public health translation.

Therefore, the aim of this study was to examine associations between intakes of *1*) a combined flavodiet score; *2*) flavonoid-rich foods and beverages; *3*) total flavonoids; and *4*) flavonoid subclasses and 3 indicators of healthy aging (namely, frailty, impaired physical function, and poor mental health) in 2 large cohorts. We hypothesized that higher intakes of flavonoid-rich foods and their bioactive flavonoid compounds would be associated with lower risk of frailty, impaired physical function, and poor mental health across both cohorts.

## Methods

### Study population

This study used data from 2 longitudinal studies: the NHS, initiated in 1976 with 121,701 female nurses aged between 30 and 55 years at recruitment [11], and the Health Professionals Follow-up Study (HPFS), which started in 1986 with a group of 51,529 male health professionals aged 40 to 75 years at recruitment [12]. Participants in both cohorts filled out questionnaires pertaining to their lifestyle and medical history at baseline and every subsequent 2 y, supplying current lifestyle data and information on any newly developed diseases.

**FIGURE 1:**
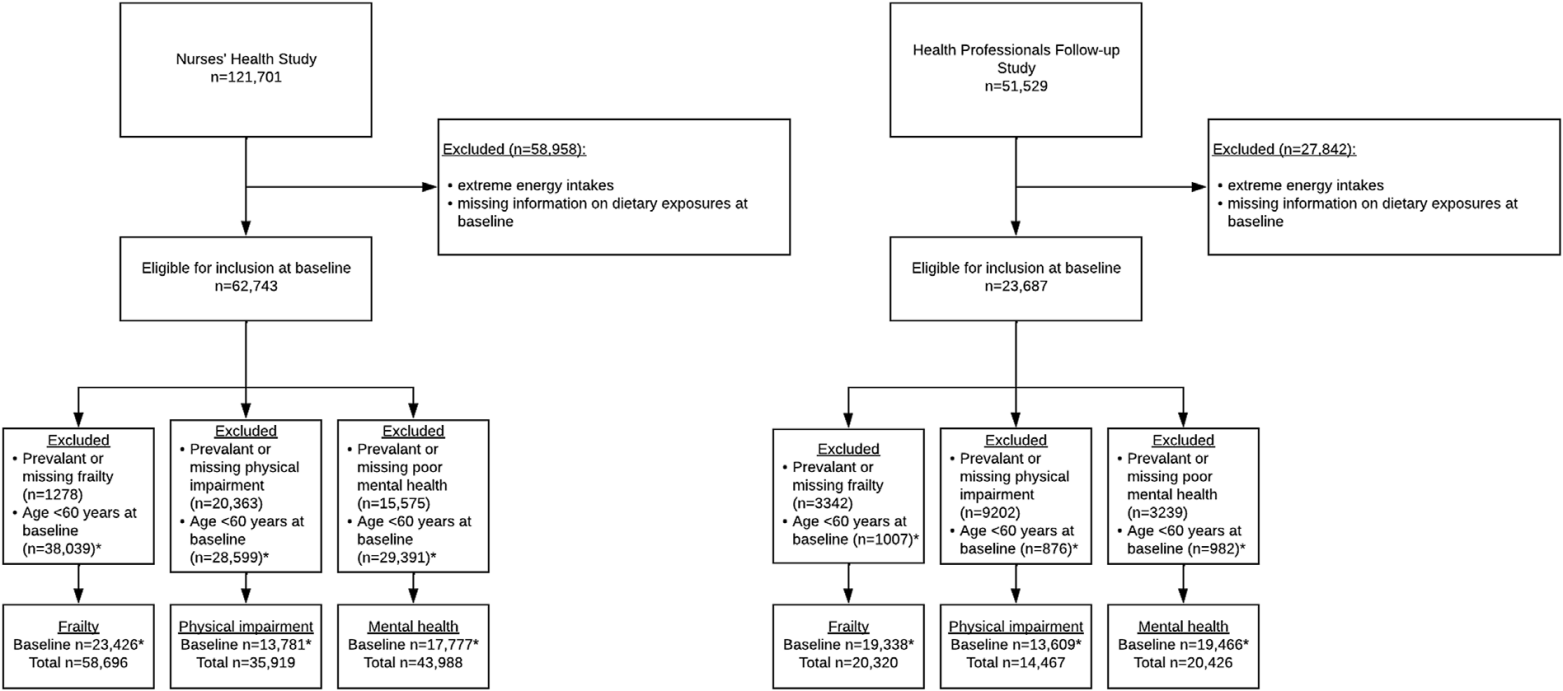
Consort flow diagram for the Nurses’ Health Study and the Health Professionals Follow-up Study. ∗Participants younger than 60 y at baseline entered the study only at the wave in which their age exceeded 60 y.

Based on the questionnaire cycles in which the outcome data were collected, for analyses in this study participants from the NHS were followed up from 1990 (considered baseline) to 2014 whereas participants from the HPFS were followed from 2006 (considered baseline) to 2018. We excluded participants with extreme energy intakes (<800 or >4200 kcal/d for males and <500 or >3500 kcal/d for females) and participants with missing information on dietary exposures at the analytic baseline. Furthermore, participants were only included in analyses when they reached 60 y or older; that is, participants <60 y at baseline only entered the study at the wave in which their age exceeded 60 y. The final analysis included 62,743 females and 23,687 males at each study’s respective baseline (Figure 1).

The study protocol was approved by the institutional review boards (IRBs) of the Brigham and Women’s Hospital (Nurses’ Health Study; IRB Protocol Number: 1999P011114) and Harvard T.H. Chan School of Public Health (Health Professionals Follow-up Study; IRB Protocol Number: 10162). Completion and return of study questionnaires implied informed consent of the participants.

### Dietary assessment

Participants of both cohorts filled out validated, semiquantitative food frequency questionnaires (FFQs) at baseline, with subsequent follow-ups every 4 years. These questionnaires asked participants to report the average frequency and portion size of their consumption of certain food and drink items over the past year, with responses ranging from never or less than once a month to 6 or more times per day. Frequencies of consumption of flavonoid-rich foods were recorded as number of servings per day, week, or month. The consumption of flavonoid-rich foods/beverages, contributing to >1% of total flavonoid intake in both the NHS and HPFS at all data collection points (i.e., tea, apples, oranges, grapefruits, blueberries, strawberries, and red wine) was aggregated to formulate a flavodiet score by summing intakes in serves per day [10]. Both time-updated intakes and change in intake of the flavodiet score and each of its individual components were examined. For the flavodiet score, participants were divided into 7 categories: 3 increase categories (increase of 1–3.9 servings/wk; increase of 4–6.9 servings/wk; and increase of ≥7 servings/wk); 3 decrease categories (decrease of 1–3.9 servings/wk; decrease of 4–6.9 servings/wk; decrease of ≥7 servings/wk), and 1 reference category (no change; ±<1 serving/wk). Similarly, when the exposure of interest was change in intakes of flavonoid-rich foods, participants were divided into 7 categories: 3 increase categories (increase of 0.5–0.99 servings/wk; increase of 1–1.99 servings/wk; increase of ≥2 servings/wk); 3 decrease categories (decrease of 0.5–0.99 servings/wk; decrease of 1–1.99 servings/wk; decrease of ≥2 servings/wk), and 1 reference category (no change; ±<0.49 servings per week). From the FFQ, time-updated intakes (in milligrams per day) of total flavonoids and flavonoid subclasses were estimated as described previously [13]. Briefly, a comprehensive database for assessing intakes of flavonoid subclasses was developed using the USDA flavonoid and proanthocyanidin databases as primary sources, supplemented with European (EuroFIR) data and other literature. Intakes of 6 main flavonoid subclasses commonly consumed in the United States diet were calculated by assigning flavonoid values to foods in the FFQ, imputing values for similar foods when necessary, and summing the consumption frequency multiplied by flavonoid content, with total flavonoid intakes derived as the sum of these subclasses. Flavonoid subclasses included the following: *1*) flavonols; *2*) flavan-3-ols monomers; *3*) flavan-3-ol polymers (including proanthocyanidins, theaflavins, and thearubigins); *4*) anthocyanins; *5*) flavanones; and *6*) flavones. The validity and reproducibility of the FFQs have been previously documented; the correlations between major dietary sources of flavonoids, specifically apples, tea, and wine, as measured by diet-records and FFQs, were 0.70, 0.77, and 0.83, respectively [14,15]. Furthermore, these FFQ-derived intakes of total flavonoids and flavonoid subclasses (except for flavones) have shown good validity and reproducibility in these cohorts [16].

### Outcomes

#### Frailty

Participants completed a Medical Outcomes Study Short-Form Health Survey (SF-36) [17], every 4 years, from 1992 to 2012 in the NHS and from 2008 to 2016 in the HPFS [18]. Frailty was defined as having ≥3 of the following 5 self-reported criteria from the FRAIL(Fatigue, Resistance, Aerobic capabity, Illness and Loss of weight) scale: fatigue, poor strength, reduced aerobic capacity, having ≥5 chronic illnesses, and weight loss ≥5%. As described previously [19], the first 3 criteria were assessed using questions from the SF-36. When frailty was the outcome of interest, we excluded participants identified as frail (≥3 frailty criteria) upon entry into the study.

#### Physical impairment

Physical function was assessed on the basis of 10 questions within the SF-36, which inquired about physical limitations in performing various moderate and vigorous activities as described previously and referred to as the PF-10 [20]. In brief, each question had the same 3 response choices: “Yes, limited a lot,” “Yes, limited a little,” or “No, not limited at all.” No impairment of physical function was defined as reporting “No, not limited at all” on moderate activities and no more than “Yes, limited a little” on vigorous activities. Participants who reported “Yes, limited a lot” for any of the activities were considered to have limitations in physical function. From these 10 questions, a physical function score (PFS) was calculated, ranging from 10 to 30. The raw score was then transformed to a 100-point scale; participants were classified as being physically impaired if they had a PFS score of <80 as done previously [20]. When physical function was the outcome of interest, we excluded participants identified as being physically impaired (PFS <80) upon entry into the study.

#### Poor mental health

Mental health was assessed from 5 questions (frequently referred to as the MF-5) in the SF-36 in 1992, 1996, and 2000 in the NHS and then by the 10 questions in the Center for Epidemiologic Studies Depression in 2004 in the NHS and 15 questions in the Geriatric Depression Scale 15 in 2008 and 2012 in the NHS and 2008, 2012, and 2016 in the HPFS. Poor mental health was defined as an MF-5 score ≤52, a Center for Epidemiologic Studies Depression 10 score ≥10, or a Geriatric Depression Scale 15 score ≥6 as done previously [21]. When poor mental health was the outcome of interest, we excluded participants identified as having poor mental health upon entry into the study.

### Assessment of covariates

Every 2 years, participants provided information on their demographic characteristics (such as age, race/ethnic group, marital status, menopausal status, and body measurements), lifestyle behaviors (including physical activity levels, smoking habits, and use of aspirin, multivitamins, postmenopausal hormone therapy, diuretics, β-blockers, calcium channel blockers, angiotensin-converting enzyme inhibitors, other antihypertensives, statins, insulin, and oral hypoglycemic medications), family medical history (myocardial infarction, type 2 diabetes, and cancer), and any recent diagnoses of diseases for themselves (such as myocardial infarction, stroke, type 2 diabetes, cancer, hypertension, and hypercholesterolemia) through a questionnaire. Physical activity was quantified in terms of metabolic equivalent tasks, translating to energy expended per hour each week, as described previously [22]. Dietary intakes of alcohol, total energy, meat, nuts, saturated fat, polyunsaturated fat, trans fat, cereal fiber, and soft drinks were assessed and updated every 4 y using the FFQ.

### Statistical analysis

We calculated person years of follow-up from the date of return of the questionnaire completed upon entry into the analytic cohort to the date of the respective outcome, death, or loss to follow-up, whichever came first. We used time-dependent Cox proportional hazards regression models to estimate the hazard ratios (HRs) and 95% CIs of incident frailty, physical impairment, and poor mental health, separately. Owing to the timing of the questionnaires (with exposures and outcomes assessed in alternate questionnaires sent out every 2 y), there was a 2-y lag between exposure and outcome assessments. Proportional hazards assumptions were checked for the 2 main exposures (flavodiet score and total flavonoid intakes) with no violations found. For time-updated intakes of each exposure (i.e., flavodiet score, and flavonoid-rich foods, total flavonoids, and flavonoid subclasses), participants were categorized into quartiles or quintiles as appropriate based on the distribution of the respective exposure. The 4-y changes in consumption of the flavodiet score and individual flavonoid-rich foods (change categories described earlier) were modeled as time varying exposures as done previously [10]. Participants contributed risk time to the corresponding change category for each interval, based on their dietary intake during that period. Hazard ratios and 95% CIs were also estimated for a 3 servings/d increase in flavodiet score. For flavonoid-rich foods, HRs and 95% CIs were also estimated for a 3.5-servings/week increase in intake, representing an additional serving every second day, except for tea intake, which was estimated for an increase of 7 servings/wk, representing an additional serving every day.

When exposures were modeled as time-updated intakes, the following adjustment strategy was used: model 1 adjusted for age (calendar year) and questionnaire cycle as a proxy for calendar time; model 2 adjusted for age; questionnaire cycle; ethnicity (White and other); smoking status (never, former, and current); marital status (married and unmarried); menopausal status [premenopausal and postmenopausal, never user of hormone therapy (HT), postmenopausal and current user of HT, and postmenopausal and past user of HT]; a family history of myocardial infarction, type 2 diabetes, and cancer (all yes and no); multivitamin use (yes/no); use of aspirin (yes/no); use of other medications (diuretics, β-blockers, calcium channel blockers, angiotensin-converting enzyme inhibitors, other antihypertensives, statins, and insulin or oral hypoglycemic medications); a history of hypertension, hypercholesterolemia, type 2 diabetes, myocardial infarction, and stroke (all yes and no); physical activity (quintiles); BMI (<23, 23–24.9, 25–29.9, 30–34.9, and >35 kg/m^2^); and intakes of alcohol (0, 0.1–4.9, 5–14.9, 15–29.9, and ≥30 g/d), total energy, meat, nuts, saturated fat, polyunsaturated fat, trans fat, cereal fiber, and soft drink (quintiles). When exposures were modeled as change in intakes, intakes of the exposure variable of interest were added to both models and change in smoking status (never to never, never to current, former to former, former to current, current to former, current to current, or missing indicator); change in physical activity; change in BMI; and change in intakes of alcohol, total energy, meat, nuts, saturated fat, polyunsaturated fat, trans fat, cereal fiber, and soft drink were added to model 2. All covariates are time updated (except where change in the covariate is modeled) and are carried forward from previous observations for missing values except for diet where values were carried forward only once before censoring the follow-up for missing values.

We also conducted 3 sensitivity analyses: *1*) rather than censoring upon death, death was included as an event for each of the 3 outcomes; *2*) we censored NHS participants at 12 y of follow-up to explore whether the difference in length of follow-up could explain the different associations observed between males and females; and *3*) we left-truncated the analyses so that participants of the NHS only entered the study at 70 y of age to explore whether differences in age at baseline could explain the different associations observed between males and females.

All analyses were carried out in SAS version 9.2 (SAS Institute). Statistical tests were 2 sided and a *P* value of <0.05 was considered statistically significant.

## Results

Among females, we documented 11,369 incident cases of frailty, 22,419 incident cases of physical impairment, and 8944 cases of poor mental health of 58,696, 35,919, and 43,988 females, respectively, over 24 y (i.e., 5 waves) of follow-up. The median follow-up times were 186 mo for frailty, 116 mo for physical impairment, and 188 mo for poor mental health. Among males, we documented 1957 incident cases of frailty, 4165 incident cases of physical impairment and 1669 cases of poor mental health of 20,320, 14,467, and 20,426 males, respectively, over 12 y (i.e., 2 waves) of follow-up. The median follow-up times were 141 mo for frailty, 133 mo for physical impairment, and 141 mo for poor mental health.

### Baseline characteristics results

Across the 3 NHS subpopulations, participants with the highest baseline flavodiet scores tended to be more physically active, have never smoked, have a lower alcohol but a higher total energy intake, and were more likely to have high cholesterol and to take a multivitamin than those with the lowest flavodiet scores (Table 1). Similar patterns were seen across the 3 HPFS subpopulations, except that participants with the highest baseline flavodiet scores tended to have a lower body weight and BMI, to have a higher intake of alcohol, and were less likely to be hypertensive, have high cholesterol, have a history of type 2 diabetes, and take medications including aspirin (Table 1).

**TABLE 1:** Baseline characteristics of the different populations.

Nurses’ Health Study (1990)						
Outcome	Frailty (*n* = 23,426)		Impaired physical function (*n* = 13,781)		Poor mental health (*n* = 17,777)	
	Flavodiet score Quintile 1 (*n* = 4406)	Flavodiet score Quintile 5 (*n* = 4427)	Flavodiet score Quintile 1 (*n* = 2838)	Flavodiet score Quintile 5 (*n* = 2478)	Flavodiet score Quintile 1 (*n* = 3418)	Flavodiet score Quintile 5 (*n* = 3419)
Flavodiet score (serves/wk)	1.8 (1.0)	26.3 (8.3)	2.2 (1.1)	26.8 (8.2)	2.0 (1.0)	26.2 (8.2)
Age (y)	64.0 (2.6)	64.2 (2.7)	63.8 (2.6)	64.1 (2.7)	63.9 (2.6)	64.1 (2.7)
Follow-up time (mo)	199 (79)	211 (76)	136 (72)	145 (74)	212 (72)	220 (70)
BMI (kg/m^2^)	25.6 (4.7)	25.5 (4.5)	24.5 (3.8)	24.6 (3.7)	25.6 (4.7)	25.4 (4.4)
Weight (kg)	68.3 (13.4)	68.1 (12.8)	65.4 (10.8)	65.4 (10.8)	68.5 (13.2)	67.9 (12.7)
Physical activity (MET h/wk)	13.4 (18.6)	17.3 (21.1)	16.0 (22.0)	19.3 (22.8)	13.8 (20.0)	17.7 (20.2)
Alcohol intake (g/d)	6.5 (12.2)	4.5 (9.2)	7.1 (12.3)	5.1 (9.7)	6.3 (11.6)	4.6 (9.1)
Energy intake (calories/d)	1559 (475)	1852 (526)	1551 (463)	1853 (523)	1557 (466)	1848 (522)
Ethnicity (% White)	98.3	98.7	98.1	98.4	98.6	98.8
Marital status (% married)	73.0	75.5	76.1	78.0	76.2	78.1
Never smoker (%)	37.5	47.7	40.4	49.8	40.0	48.0
Former smoker (%)	37.7	41.0	36.6	39.5	39.0	42.2
Current smoker (%)	24.6	11.0	22.7	10.3	20.7	9.6
Premenopausal (%)	0.02	0.02	0.0	0.0	0.0	0.0
HRT never user (%)	42.3	41.2	42.7	42.4	41.2	41.4
HRT former user (%)	28.5	27.3	27.3	26.5	28.1	26.9
HRT current user (%)	24.6	27.3	25.4	27.1	26.1	27.9
Missing HRT use (%)	4.6	4.1	4.5	4.0	4.6	3.8
Hypertension (%)	41.6	41.0	34.1	34.9	40.3	39.3
High cholesterol level (%)	46.6	47.9	43.6	46.2	46.8	47.9
Type 2 diabetes (%)	5.9	7.0	3.9	5.0	5.6	5.7
History of MI (%)	2.7	2.8	1.7	1.3	2.2	2.3
History of stroke (%)	1.3	1.2	0.6	0.6	0.9	0.7
Family history of MI (%)	23.8	25.4	22.2	25.5	24.0	25.5
Family history of diabetes (%)	30.8	31.8	29.3	29.5	30.9	31.1
Family history of cancer (%)	60.0	60.7	60.2	61.0	61.2	62.2
Aspirin (%)	47.3	47.0	48.6	49.0	47.3	48.1
Multivitamin (%)	37.4	40.2	36.4	39.8	36.7	39.3
Other medication (%)	44.8	45.1	39.1	39.2	45.2	45.3
Meat intake (serves/d)	1.1 (0.8)	1.1 (0.8)	1.0 (0.8)	1.1 (0.8)	1.1 (0.8)	1.1 (0.8)
Nuts intake (serves/d)	0.2 (0.3)	0.2 (0.4)	0.2 (0.3)	0.2 (0.4)	0.2 (0.3)	0.2 (0.4)
Saturated fat intake (g/d)	19.2 (8.2)	20.3 (8.0)	18.7 (8.0)	20.0 (8.0)	19.1 (8.0)	20.2 (8.1)
Polyunsaturated fat intake (g/d)	10.2 (4.6)	12.0 (4.9)	10.2 (4.5)	12.0 (4.8)	10.3 (4.6)	12.0 (4.9)
Trans fat intake (g/d)	2.8 (1.5)	2.8 (1.5)	2.7 (1.4)	2.8 (1.4)	2.8 (1.5)	2.8 (1.5)
Cereal fiber intake (g/d)	4.9 (3.6)	6.4 (4.0)	5.0 (3.4)	6.6 (4.1)	4.9 (3.4)	6.5 (4.0)
Soft drink intake (serves/d)	0.2 (0.5)	0.2 (0.4)	0.2 (0.5)	0.2 (0.4)	0.2 (0.5)	0.2 (0.4)

Health Professionals Follow-up Study (2006)						

Outcome	Frailty (*n* = 19,338)		Impaired physical function (*n* = 13,609)		Poor mental health (*n* = 19,465)	
	Flavodiet score Quintile 1 (*n* = 3786)	Flavodiet score Quintile 5 (*n* = 3944)	Flavodiet score Quintile 1 (*n* = 2700)	Flavodiet score Quintile 5 (*n* = 2781)	Flavodiet score Quintile 1 (*n* = 3831)	Flavodiet score Quintile 5 (*n* = 3918)
Flavodiet score (serves/wk)	2.2 (1.2)	27.3 (9.7)	2.4 (1.3)	28 (10)	2.2 (1.2)	27.2 (9.7)
Age (y)	70.9 (7.8)	71.0 (7.4)	69.0 (6.9)	69.5 (6.6)	71.2 (7.8)	71.1 (7.4)
Follow-up time (mo)	114 (39)	119 (37)	112 (36)	116 (35)	113 (40)	119 (37)
BMI (kg/m^2^)	26.5 (3.8)	25.6 (3.6)	26.2 (3.6)	25.4 (3.2)	26.6 (3.9)	25.6 (3.5)
Weight (kg)	84.5 (13.7)	81.9 (12.6)	84.1 (12.9)	81.2 (11.7)	84.9 (14.1)	82.0 (12.5)
Physical activity (MET h/wk)	34.5 (32.0)	47.3 (36.8)	37.4 (32.8)	51.1 (37.7)	34.0 (32.1)	47.3 (36.9)
Alcohol intake (g/d)	11.0 (16.2)	18.5 (20.1)	11.3 (16.2)	19.1 (19.8)	11.0 (16.1)	18.4 (20.1)
Energy intake (calories/d)	1797 (567)	2243 (620)	1810 (576)	2247 (615)	1795 (566)	2244 (622)
Ethnicity (% White)	96.4	96.0	96.0	96.1	96.3	96.0
Marital status (% married)	85.3	88.2	87.3	88.7	85.4	88.1
Never smoker (%)	47.8	51.6	49.6	53.4	46.8	51.4
Former smoker (%)	47.1	46.9	45.4	45.4	48.1	47.1
Current smoker (%)	5.1	1.5	5.0	1.2	5.1	1.5
Hypertension (%)	56.3	51.6	52.4	47.8	57.7	52.0
High cholesterol level (%)	64.8	61.6	64.0	60.1	64.9	61.6
Type 2 diabetes (%)	10.3	8.8	8.8	7.3	11.4	9.3
History of MI (%)	9.7	8.4	7.5	6.6	10.7	8.6
History of stroke (%)	2.5	2.8	1.6	1.6	2.7	2.7
Family history of MI (%)	36.8	36.1	35.5	34.5	37.6	35.7
Family history of diabetes (%)	16.1	15.5	15.7	14.8	16.8	15.6
Family history of cancer (%)	39.0	38.8	38.0	38.0	39.4	38.6
Aspirin (%)	60.1	62.3	60.6	61.2	60.3	62.2
Multivitamin (%)	65.7	74.3	65.9	74.3	66.0	74.6
Other medication (%)	64.4	61.3	62.1	58.1	65.8	61.4
Meat intake (serves/d)	1.1 (0.7)	0.9 (0.7)	1.1 (0.8)	0.9 (0.7)	1.1 (0.7)	0.9 (0.7)
Nuts intake (serves/d)	0.7 (0.9)	1.1 (1.1)	0.7 (0.9)	1.1 (1.2)	0.7 (0.9)	1.1 (1.1)
Saturated fat intake (g/d)	21.7 (9.8)	22.5 (9.8)	21.6 (9.7)	22.4 (9.5)	21.7 (9.)	22.6 (9.8)
Polyunsaturated fat intake (g/d)	13.9 (6.4)	18.1 (8.8)	14.2 (6.6)	18.3 (8.9)	13.9 (6.3)	18.1 (8.8)
Trans fat intake (g/d)	1.8 (1.0)	1.6 (0.9)	1.8 (1.0)	1.6 (0.8)	1.8 (1.0)	1.6 (0.9)
Cereal fiber intake (g/d)	6.4 (3.7)	8.2 (4.5)	6.4 (3.9)	8.3 (4.5)	6.4 (3.8)	8.2 (4.5)
Soft drink intake (serves/d)	0.4 (0.7)	0.2 (0.5)	0.4 (0.7)	0.2 (0.5)	0.3 (0.7)	0.2 (0.5)

Continuous variables are expressed as mean (SD), whereas binary variables are expressed as %.

Abbreviations: HRT, hormone-replacement therapy; MET, metabolic equivalent of task; MI, myocardial infarction.

### Time-updated flavodiet and flavonoid intake results

In the NHS, the highest flavodiet scores, compared with the lowest, were associated with 15% lower risk of developing frailty (HR_Q5vsQ1_: 0.85; 95% CI: 0.80, 0.90), 12% lower risk of developing impaired physical function (HR_Q5vsQ1_: 0.88; 95% CI: 0.84, 0.91), and 12% lower risk of developing poor mental health (HR_Q5vsQ1_: 0.88; 95% CI: 0.82, 0.94) after multivariable adjustments (model 2, Table 2). For total flavonoids, the highest compared with the lowest intakes, were associated with 14% lower risk of frailty (HR_Q5vsQ1_: 0.86; 95% CI: 0.80, 0.92), 11% lower risk of developing impaired physical function (HR_Q5vsQ1_: 0.89; 95% CI: 0.85, 0.93), and 11% lower risk of developing poor mental health (HR_Q5vsQ1_: 0.89; 95% CI: 0.83, 0.96) after multivariable adjustments (model 2, Table 2). Fewer associations were observed in the HPFS; moderate (quintile 4) intakes of total flavonoids were associated with lower risk of impaired physical function (HR_Q5vsQ1_: 0.88; 95% CI: 0.79, 0.99), although the highest flavodiet score intakes were associated with lower risk of poor mental health (HR_Q5vsQ1_: 0.82; 95% CI: 0.69, 0.97) (model 2; Table 2).

**TABLE 2:** Associations between time-updated flavodiet score and total flavonoid intakes and healthy aging domains in the Nurses’ Health Study I and Health Professionals Follow-up Study.

	Exposure quintiles					*P*-trend
	Q1	Q2	Q3	Q4	Q5	
Nurses’ Health Study I						
Frailty						
Flavodiet score						
Intake (serving/wk)	2.0 (1.0–2.0)	5.0 (4.4–6.0)	8.5 (7.5–9.5)	13.0 (11.5–15.0)	23.5 (20.0–30.0)	
Events/py	3005/166,171	2391/164,619	2326/172,278	1891/176,733	1756/168,427	
Model 1	Ref	0.83 (0.79, 0.88)	0.78 (0.74, 0.82)	0.64 (0.61, 0.68)	0.64 (0.60, 0.89)	<0.001
Model 2	Ref	0.96 (0.91, 1.02)	0.95 (0.89, 1.00)	0.87 (0.82, 0.93)	0.85 (0.80, 0.90)	<0.001
Total flavonoids						
Intake (mg/d)	96.2 (70.0–115.9)	171.5 (152.2–190.5)	256.3 (232.1–285.2)	402.1 (360.4–450.5)	872.8 (638.9–998.7)	
Events/py	2692/151,028	2429/168,941	2313/176,548	2073/176,853	1862/174,859	
Model 1	Ref	0.82 (0.78, 0.87)	0.76 (0.72, 0.81)	0.69 (0.65, 0.73)	0.64 (0.60, 0.68)	<0.001
Model 2	Ref	0.94 (0.89, 1.00)	0.94 (0.89, 1.00)	0.89 (0.84, 0.95)	0.86 (0.80, 0.92)	<0.001
Impaired physical function						
Flavodiet score						
Intake (serving/wk)	2.0 (1.5–3.0)	6.0 (5.0–6.9)	9.5 (8.5–10.0)	14.0 (12.5–16.0)	24.0 (20.9–30.5)	
Events/py	5255/72,312	4746/75,512	4257/71,938	4138/74,525	4023/74,850	
Model 1	Ref	0.87 (0.84, 0.90)	0.83 (0.80, 0.87)	0.77 (0.74, 0.81)	0.76 (0.73, 0.79)	<0.001
Model 2	Ref	0.93 (0.90, 0.97)	0.92 (0.88, 0.96)	0.89 (0.85, 0.93)	0.88 (0.84, 0.91)	<0.001
Total flavonoids						
Intake (mg/d)	97.2 (71.5–116.0)	172.4 (152.8–191.0)	257.0 (233.0–285.4)	402.5 (359.4–450.9)	867.0 (616.7–989.4)	
Events/py	4055/57,071	4552/70,618	4687/78,303	4633/81,051	4492/82,094	
Model 1	Ref	0.92 (0.88, 0.96)	0.85 (0.82, 0.89)	0.81 (0.78, 0.85)	0.78 (0.75, 0.81)	<0.001
Model 2	Ref	0.98 (0.93, 1.01)	0.94 (0.90, 0.98)	0.91 (0.87, 0.95)	0.89 (0.85, 0.93)	<0.001
Poor mental health						
Flavodiet score						
Intake (serving/wk)	2.0 (1.0–2.9)	5.5 (4.5–6.4)	9.0 (8.0–9.5)	13.4 (12.0–15.0)	23.5 (20.1–29.5)	
Events/py	2317/132,212	1860/131,477	1739/130,441	1492/129,152	1536/129,399	
Model 1	Ref	0.85 (0.80, 0.90)	0.82 (0.77, 0.87)	0.73 (0.68, 0.78)	0.76 (0.71, 0.81)	<0.001
Model 2	Ref	0.94 (0.88, 1.00)	0.93 (0.88, 1.00)	0.87 (0.81, 0.93)	0.88 (0.82, 0.94)	0.001
Total flavonoids						
Intake (mg/d)	96.9 (71.1–116.3)	171.9 (152.9–190.9)	256.2 (232.3–285.1)	401.0 (359.8–450.2)	873.5 (633.2–994.4)	
Events/py	1893/114,096	1779/130,524	1834/136,857	1755/137,003	1683/134,201	
Model 1	Ref	0.83 (0.78, 0.89)	0.83 (0.78, 0.89)	0.81 (0.76, 0.86)	0.79 (0.74, 0.85)	<0.001
Model 2	Ref	0.90 (0.84, 0.96)	0.93 (0.87, 1.00)	0.90 (0.84, 0.97)	0.89 (0.83, 0.96)	0.038
Health Professionals Follow-up Study						
Frailty						
Flavodiet score						
Intake (serving/wk)	2.3 (1.5–3.3)	6.0 (5.0–7.0)	9.5 (8.5–10.3)	14.0 (12.5–15.7)	24.0 (20.5–30.5)	
Events/py	481/39,044	416/39,701	357/39,369	384/40,317	319/39,940	
Model 1	Ref	0.88 (0.76, 1.00)	0.77 (0.67, 0.89)	0.80 (0.70, 0.92)	0.71 (0.61, 0.82)	0.003
Model 2	Ref	1.01 (0.88, 1.16)	0.94 (0.81, 1.08)	1.09 (0.94, 1.26)	0.98 (0.84, 1.15)	0.241
Total flavonoids						
Intake (mg/d)	130.0 (98.9–154.1)	220.4 (199.2–240.8)	309.9 (285.1–336.2)	432.2 (396.5–471.4)	696.4 (584.5–931.9)	
Events/py	427/34,616	418/38,941	392/40,732	399/41,733	321/42,350	
Model 1	Ref	0.86 (0.75, 0.99)	0.79 (0.69, 0.91)	0.80 (0.69, 0.92)	0.64 (0.55, 0.74)	<0.001
Model 2	Ref	1.01 (0.87, 1.17)	1.00 (0.86, 1.17)	1.06 (0.91, 1.24)	0.93 (0.78, 1.10)	0.427
Impaired physical function						
Flavodiet score						
Intake (serving/wk)	2.5 (1.5–3.5)	6.3 (5.3–7.0)	9.8 (8.8–10.7)	14.5 (13.0–16.0)	24.5 (20.9–31.0)	
Events/py	981/26,844	838/26,468	823/28,497	802/27,242	721/27,668	
Model 1	Ref	0.87 (0.79, 0.96)	0.78 (0.71, 0.86)	0.78 (0.71, 0.86)	0.74 (0.67, 0.81)	<0.001
Model 2	Ref	0.95 (0.87, 1.05)	0.90 (0.82, 1.00)	0.97 (0.87, 1.07)	0.93 (0.84, 1.04)	0.949
Total flavonoids						
Intake (mg/d)	132.5 (100.5–154.7)	221.3 (200.1–241.5)	310.7 (285.6–337.3)	431.6 (396.3–471.6)	697.7 (584.4–943.0)	
Events/py	806/21,947	851/26,154	882/28,253	826/29,820	800/30,546	
Model 1	Ref	0.89 (0.80, 0.98)	0.84 (0.76, 0.92)	0.74 (0.67, 0.82)	0.72 (0.65, 0.79)	<0.001
Model 2	Ref	0.96 (0.87, 1.07)	0.95 (0.86, 1.06)	0.88 (0.79, 0.99)	0.92 (0.82, 1.04)	0.166
Poor mental health						
Flavodiet score						
Intake (serving/wk)	2.3 (1.5–3.3)	6.0 (5.0–7.0)	9.5 (8.5–10.3)	14.0 (12.5–15.7)	24.0 (20.5–30.5)	
Events/py	420/39,210	315/39,858	334/39,441	323/40,495	277/39,859	
Model 1	Ref	0.77 (0.66, 0.89)	0.83 (0.71, 0.96)	0.76 (0.66, 0.88)	0.68 (0.59, 0.80)	<0.001
Model 2	Ref	0.85 (0.73, 0.99)	0.94 (0.81, 1.09)	0.90 (0.77, 1.06)	0.82 (0.69, 0.97)	0.0658
Total flavonoids						
Intake (mg/d)	129.9 (99.1–154.1)	220.6 (199.4–241.1)	309.2 (285.0–335.8)	432.2 (396.5–471.6)	693.4 (584.3–928.4)	
Events/py	366/34,834	323/39,099	339/40,740	316/42,027	325/42,163	
Model 1	Ref	0.80 (0.69, 0.93)	0.83 (0.71, 0.96)	0.74 (0.63, 0.86)	0.76 (0.65, 0.89)	0.002
Model 2	Ref	0.88 (0.75, 1.03)	0.94 (0.80, 1.10)	0.84 (0.71, 1.00)	0.88 (0.73, 1.06)	0.271

Hazard ratios (95% CI) for frailty, physical impairment, and poor mental health during 24 y of follow-up, obtained from Cox proportional hazards models. Model 1 adjusted for age and questionnaire cycle; model 2 adjusted for age; questionnaire cycle; ethnicity; smoking status; marital status; menopausal status; a family history of myocardial infarction, diabetes, and cancer; multivitamin use; use of other medications; a history of hypertension, hypercholesterolemia, diabetes, myocardial infarction, and stroke; physical activity; BMI; and intakes of alcohol, total energy, meat, nuts, saturated fat, polyunsaturated fat, trans fat, cereal fiber, and soft drink. Intakes are reported as median (p25–p75).

Abbreviation: py, person years.

In the NHS, the highest intakes of tea, red wine, blueberries, apples, and oranges/orange juice were associated with 11%–21% lower risk of developing frailty, compared with the lowest intakes (model 2, Table 3). Similarly, the highest intakes of red wine, blueberries, apples, strawberries, and oranges/orange juice were associated with 4%–14% lower risk of physical impairment although the highest intakes of apples, strawberries, oranges/orange juice, and grapefruit/grapefruit juice were associated with 10%–15% lower risk of poor mental health, when compared with the lowest intakes (model 2, Table 3). In the HPFS, the highest intakes of tea and blueberries and moderate average intakes of red wine (quartile 3) were associated with 14% (HR_Q5vsQ1_: 0.86; 95% CI: 0.76, 0.98), 15% (HR_Q5vsQ1_: 0.85; 95% CI: 0.74, 0.98), and 29% (HR_Q5vsQ1_: 0.71; 95% CI: 0.62, 0.80) lower risk of poor mental health, respectively (model 2, Supplemental Table 1). There were no associations between intakes of any of the flavonoid-rich foods and beverages and either frailty or impaired physical function in the HPFS.

**TABLE 3:** Associations between time-updated flavonoid-rich food intakes and healthy aging domains in the Nurses’ Health Study I.

	Quartiles of intake				*P*-trend
	Q1	Q2	Q3	Q4	
Frailty					
Tea					
Intake (servings/wk)	0 (0–0)	0.5 (0.5–1.00)	3.0 (3.0–5.5)	7 (7–17.5)	
Events/py	3782/250,810	2399/182,748	2408/180,332	2780/234,339	
Model 1	Ref.	0.90 (0.86, 0.95)	0.81 (0.77, 0.85)	0.81 (0.77, 0.85)	<0.001
Model 2	Ref.	0.93 (0.88, 0.98)	0.88 (0.84, 0.93)	0.89 (0.85, 0.94)	0.013
Red wine					
Intake (servings/wk)	0 (0–0)	0.5 (0.5–0.5)	1.0 (1.0–1.0)	3.0 (3.0–5.5)	
Events/py	9176/597,981	1065/115,503	400/46,138	728/88,606	
Model 1	Ref.	0.67 (0.63, 0.72)	0.61 (0.56, 0.68)	0.50 (0.47, 0.54)	<0.001
Model 2	Ref.	0.89 (0.83, 0.96)	0.89 (0.80, 0.99)	0.81 (0.74, 0.89)	0.002
Blueberry					
Intake (servings/wk)	0 (0–0)	0.5 (0.5–0.5)	1.0 (1.0–1.0)	3.0 (3.0–3.0)	
Events/py	6258/432,381	2637/222,811	1380/118,309	1094/74,728	
Model 1	Ref.	0.80 (0.77, 0.84)	0.73 (0.69, 0.78)	0.69 (0.65, 0.74)	<0.001
Model 2	Ref.	0.90 (0.86, 0.94)	0.88 (0.82, 0.93)	0.89 (0.83, 0.95)	0.002
Apple					
Intake (servings/wk)	0 (0–0)	0.5 (0.5–0.5)	3.0 (1.0–3.0)	7.0 (5.5–7.0)	
Events/py	2474/122,755	3175/212,213	4722/407,396	998/105,865	
Model 1	Ref.	0.86 (0.82, 0.91)	0.69 (0.66, 0.73)	0.63 (0.58, 0.67)	<0.001
Model 2	Ref.	0.94 (0.89, 0.99)	0.86 (0.81, 0.90)	0.83 (0.77, 0.90)	<0.001
Strawberry					
Intake (servings/wk)	0 (0–0)	0.5 (0.5–0.5)	1.0 (1.0–1.0)	3.0 (3.0–3.0)	
Events/py	3429/206,537	4038/321,027	2487/214,307	1415/106,357	
Model 1	Ref.	0.89 (0.85, 0.94)	0.85 (0.80, 0.89)	0.86 (0.81, 0.92)	<0.001
Model 2	Ref.	0.94 (0.90, 0.99)	0.93 (0.88, 0.98)	0.98 (0.92, 1.05)	0.503
Orange					
Intake (servings/wk)	0 (0–0)	0.5 (0.5–0.5)	1.0 (1.0–1.0)	3.0 (3.0–5.5)	
Events/py	4343/251,460	2896/230,728	1714/151,039	2416/215,001	
Model 1	Ref.	0.89 (0.85, 0.93)	0.82 (0.78, 0.87)	0.79 (0.75, 0.83)	<0.001
Model 2	Ref.	0.94 (0.90, 0.98)	0.93 (0.88, 0.99)	0.92 (0.87, 0.96)	0.006
Grapefruit and grapefruit juice					
Intake (servings/wk)	0 (0–0)	0.5 (0.5–0.5)	0.5 (0.5–0.5)	1.0 (1.0–3.0)	
Events/py	8376/479,327	905/67,431	888/136,551	1200/164,920	
Model 1	Ref.	0.80 (0.74, 0.85)	0.71 (0.66, 0.76)	0.77 (0.72, 0.82)	<0.001
Model 2	Ref.	0.93 (0.87, 1.00)	0.84 (0.78, 0.90)	0.97 (0.90, 1.03)	0.819
Impaired physical function					
Tea					
Intake (servings/wk)	0 (0–0)	0.5 (0.5–1.0)	3.0 (3.0–5.5)	7 (7–17.5)	
Events/py	6753/107,875	4772/80,344	4869/76,446	6025/104,474	
Model 1	Ref.	0.96 (0.92, 0.99)	0.98 (0.94, 1.01)	0.94 (0.90, 0.97)	0.044
Model 2	Ref.	0.95 (0.92, 0.99)	0.99 (0.95, 1.03)	0.96 (0.93, 1.00)	0.441
Red wine					
Intake (servings/wk)	0 (0–0)	0.5 (0.5–0.5)	1.0 (1.0–1.0)	3.0 (3.0–5.5)	
Events/py	15,567/242,188	3178/56,134	1253/23,961	2421/46,854	
Model 1	Ref.	0.91 (0.88, 0.95)	0.83 (0.79, 0.88)	0.75 (0.72, 0.79)	<0.001
Model 2	Ref.	0.97 (0.93, 1.01)	0.92 (0.86, 0.98)	0.87 (0.83, 0.92)	<0.001
Blueberry					
Intake (servings/wk)	0 (0–0)	0.5 (0.5–0.5)	1.0 (1.0–1.0)	3.0 (3.0–3.0)	
Events/py	11,549/179,902	5956/101,801	3027/55,048	1887/32,387	
Model 1	Ref.	0.93 (0.90, 0.96)	0.85 (0.81, 0.88)	0.82 (0.78, 0.86)	<0.001
Model 2	Ref.	0.97 (0.94, 1.00)	0.90 (0.86, 0.95)	0.93 (0.88, 0.98)	0.004
Apple					
Intake (servings/wk)	0.5 (0–0.5)	1.0 (1.0–1.0)	3.0 (3.0–3.0)	7.0 (5.5–7.0)	
Events/py	8922/131,077	4759/77,597	6152/107,176	2586/53,288	
Model 1	Ref.	0.93 (0.90, 0.97)	0.87 (0.84, 0.90)	0.76 (0.73, 0.80)	<0.001
Model 2	Ref.	0.97 (0.94, 1.01)	0.95 (0.92, 0.98)	0.88 (0.84, 0.92)	<0.001
Strawberry					
Intake (servings/wk)	0 (0–0)	0.5 (0.5–0.5)	1.0 (1.0–1.0)	3.0 (3.0–3.0)	
Events/py	5514/80,715	8534/141,718	5668/98,508	2703/48,197	
Model 1	Ref.	0.97 (0.94, 1.01)	0.95 (0.91, 0.98)	0.89 (0.85, 0.93)	<0.001
Model 2	Ref.	1.00 (0.97, 1.04)	0.99 (0.95, 1.02)	0.96 (0.92, 1.00)	0.006
Orange					
Intake (servings/wk)	0 (0–0)	0.5 (0.5–0.5)	1.0 (1.0–1.0)	3.0 (3.0–5.5)	
Events/py	6666/96,425	6156/101,235	4015/69,228	5582/102,250	
Model 1	Ref.	0.96 (0.92, 0.99)	0.91 (0.88, 0.95)	0.83 (0.80, 0.86)	<0.001
Model 2	Ref.	0.96 (0.93, 0.99)	0.94 (0.90, 0.98)	0.90 (0.86, 0.93)	<0.001
Grapefruit and grapefruit juice					
Intake (servings/wk)	0 (0–0)	0.5 (0.5–0.5)	1.0 (1.0–1.0)	3.0 (3.0–5.5)	
Events/py	12,519/185,434	4534/83,308	2528/48,325	2838/52,071	
Model 1	Ref.	0.92 (0.89, 0.95)	0.88 (0.84, 0.92)	0.86 (0.82, 0.89)	<0.001
Model 2	Ref.	0.98 (0.94, 1.01)	0.96 (0.92, 1.01)	0.97 (0.93, 1.01)	0.210
Poor mental health					
Tea					
Intake (servings/wk)	0 (0–0)	0.5 (0.5–1.00)	3.0 (3.0–5.5)	7 (7–17.5)	
Events/py	2875/192,429	1944/140,393	1775/139,968	2350/179,891	
Model 1	Ref.	0.94 (0.89, 0.99)	0.84 (0.79, 0.89)	0.91 (0.86, 0.96)	0.006
Model 2	Ref.	0.98 (0.92, 1.04)	0.89 (0.84, 0.95)	0.96 (0.91, 1.02)	0.103
Red wine					
Intake (servings/wk)	0 (0–0)	0.5 (0.5–0.5)	1.0 (1.0–1.0)	3.0 (3.0–5.5)	
Events/py	6872/461,176	983/87,955	348/35,604	741/67,945	
Model 1	Ref.	0.81 (0.76, 0.86)	0.70 (0.63, 0.78)	0.77 (0.71, 0.83)	<0.001
Model 2	Ref.	0.93 (0.86, 1.00)	0.84 (0.75, 0.94)	0.93 (0.85, 1.02)	0.621
Blueberry					
Intake (servings/wk)	0 (0–0)	0.5 (0.5–0.5)	1.0 (1.0–1.0)	3.0 (3.0–3.0)	
Events/py	5075/332,263	2200/172,032	1069/91,698	600/56,688	
Model 1	Ref.	0.90 (0.86, 0.95)	0.84 (0.78, 0.90)	0.81 (0.74, 0.88)	<0.001
Model 2	Ref.	0.98 (0.93, 1.03)	0.94 (0.88, 1.01)	0.94 (0.86, 1.02)	0.090
Apple					
Intake (servings/wk)	0. (0–0.)	0.5 (0.5–1.0)	3.0 (3.0–3.0)	7.0 (5.5–7.0)	
Events/py	1718/92,489	4095/296,580	2190/181,858	941/81,753	
Model 1	Ref.	0.81 (0.77, 0.86)	0.72 (0.68, 0.77)	0.71 (0.66, 0.77)	<0.001
Model 2	Ref.	0.91 (0.86, 0.96)	0.87 (0.81, 0.93)	0.87 (0.80, 0.95)	0.058
Strawberry					
Intake (servings/wk)	0 (0–0)	0.5 (0.5–0.5)	1.0 (1.0–1.0)	3.0 (3.0–3.0)	
Events/py	2783/155,989	3279/248,007	1953/167,228	929/81,456	
Model 1	Ref.	0.82 (0.78, 0.87)	0.76 (0.71, 0.80)	0.77 (0.72, 0.83)	<0.001
Model 2	Ref.	0.87 (0.83, 0.92)	0.82 (0.77, 0.87)	0.85 (0.78, 0.91)	0.003
Orange					
Intake (servings/wk)	0 (0–0)	0.5 (0.5–0.5)	1.0 (1.0–1.0)	3.0 (3.0–5.5)	
Events/py	3166/191,111	2270/175,334	1411/117,511	2097/168,725	
Model 1	Ref.	0.84 (0.79, 0.88)	0.78 (0.73, 0.83)	0.81 (0.77, 0.86)	<0.001
Model 2	Ref.	0.88 (0.83, 0.93)	0.85 (0.80, 0.91)	0.90 (0.85, 0.95)	0.151
Grapefruit and grapefruit juice					
Intake (servings/wk)	0 (0–0)	0.5 (0.5–0.5)	0.5 (0.5–1.0)	1.0 (1.0–3.0)	
Events/py	5466/365,310	772/50,218	1289/106,515	1417/130,637	
Model 1	Ref.	0.92 (0.85, 0.99)	0.88 (0.83, 0.94)	0.77 (0.72, 0.82)	<0.001
Model 2	Ref.	0.98 (0.91, 1.06)	0.95 (0.89, 1.01)	0.86 (0.80, 0.91)	<0.001

Hazard ratios (95% CI) for frailty, physical impairment and poor mental health during 24 years of follow up, obtained from Cox proportional hazards models. Model 1 adjusted for age and questionnaire cycle; Model 2 adjusted for age, questionnaire cycle, ethnicity, smoking status, marital status, menopausal status, family history of myocardial infarction, diabetes and cancer, multivitamin use, use of aspirin, use of other medications, history of hypertension, hypercholesterolemia, diabetes, myocardial infarction, and stroke, physical activity, BMI, and intakes of alcohol, total energy, meat, nuts, saturated fat, polyunsaturated fat, trans fat, cereal fiber, and soft drink. Intakes (serves per week) are reported as median [p25–p75]. Py, person years.

For each of the flavonoid subclasses, the highest intakes were associated with 9%–17% lower risk of frailty, 8%–14% lower risk of impaired physical function, and 8%–23% lower risk of poor mental health, except for the flavan-3-ol monomers, which were not associated with poor mental health (model 2, Supplemental Table 2). Fewer associations were observed in the HPFS; moderate (quintile 4) intakes of the flavan-3-ol polymers associated with 12% lower risk of developing impaired physical function while moderate (quintile 3 or 4) intakes of flavonols, flavan-3-ol polymers, and flavones and high (quintile 5) intakes of anthocyanins were associated with 16%–25% lower risk of poor mental health (Supplemental Table 3).

### Change in flavodiet and flavonoid-rich food intake results

In the NHS, compared with flavodiet scores that did not change, scores that decreased by ≥7 servings per week were associated with 18% higher risk of frailty (HR_Q5vsQ1_: 1.18; 95% CI: 1.09, 1.27) and 7% higher risk of impaired physical function (HR_Q5vsQ1_: 1.07; 95% CI: 1.02, 1.13) after multivariable adjustments (model 2, Table 4). Furthermore, a 3-servings/d increase in flavodiet score was associated with 11% lower risk of frailty (HR_Q5vsQ1_: 0.89; 95% CI: 0.84, 0.94), 7% lower risk of impaired physical function (HR_Q5vsQ1_: 0.93; 95% CI: 0.89, 0.97), and 8% lower risk of poor mental health (HR_Q5vsQ1_: 0.92; 95% CI: 0.86, 0.99) after multivariable adjustments (model 2, Table 4). Compared with intakes that remained stable, the greatest decreases in intakes of tea were associated with 7% higher risk of developing frailty, although the greatest decreases in intakes of blueberries and apples were associated with 31% and 16% higher risk of developing frailty, respectively (Table 4). Furthermore, the greatest increases in intakes of red wine were associated with 17% and 8% lower risk of frailty and impaired physical function, respectively (Table 4). When modeled as continuous variables, a 0.5-servings/d increases in intakes of red wine, apples, and oranges/orange juice were associated with a 6%–9% lower risk of frailty and a 5%–6% lower risk of impaired physical function (Table 4). Furthermore, a 0.5-servings/d increase in intakes of blueberries was associated with 12% lower risk of frailty, although the same increase in serves of strawberries was associated with 12% lower risk of poor mental health (Table 4).

**TABLE 4:** Associations between 4-y changes in intake of flavonoid-rich foods and healthy aging domains in the Nurses’ Health Study I.

	4-y change in intake levels of the flavodiet score, servings/wk							*P*-trend	Every 3 servings/d change
	Decrease			Increase					
	≥7	4–6.9	1–3.9	No change (±<1)	1–3.9	4–6.9	≥7		
Frailty									
Events/py	1577/111,904	1042/75,469	2080/148,516	2818/202,115	768/136,074	873/69,440	1211/104,710		
Model 1	1.39 (1.29, 1.49)	1.12 (1.04, 1.20)	1.07 (1.01, 1.13)	Ref.	0.97 (0.92, 1.03)	0.96 (0.89, 1.04)	0.99 (0.92, 1.06)	<0.001	0.76 (0.71, 0.80)
Model 2	1.18 (1.09, 1.27)	1.06 (0.99, 1.14)	1.06 (1.00, 1.12)	Ref.	1.01 (0.95, 1.07)	1.00 (0.92, 1.08)	1.01 (0.94, 1.08)	<0.001	0.89 (0.84, 0.94)
Impaired physical function									
Events/py	3021/47,816	2022/32,905	4053/64,474	5290/84,254	3661/60,548	1806/31,592	2566/47,549		
Model 1	1.17 (1.11, 1.23)	1.04 (0.98, 1.09)	1.02 (0.98, 1.07)	Ref.	1.00 (0.96, 1.05)	0.97 (0.92, 1.02)	0.96 (0.91, 1.00)	<0.001	0.87 (0.83, 0.90)
Model 2	1.07 (1.02, 1.13)	1.00 (0.95, 1.06)	1.01 (0.96, 1.05)	Ref.	1.01 (0.97, 1.05)	0.98 (0.93, 1.04)	0.96 (0.91, 1.00)	0.001	0.93 (0.89, 0.97)
Poor mental health									
Events/py	1215/87,099	840/58,734	1628/116,300	2320/150,364	1341/106,143	640/54,114	960/79,927		
Model 1	1.04 (0.96, 1.13)	0.96 (0.88, 1.03)	0.92 (0.87, 0.98)	Ref.	0.87 (0.82, 0.93)	0.84 (0.77, 0.92)	0.90 (0.83, 0.97)	<0.001	0.86 (0.81, 0.92)
Model 2	1.04 (0.96, 1.13)	1.01 (0.93, 1.10)	0.98 (0.92, 1.05)	Ref.	0.95 (0.89, 1.02)	0.92 (0.84, 1.01)	0.97 (0.89, 1.04)	0.018	0.92 (0.86, 0.99)

	4-y change in intake levels of flavonoid-rich foods and beverages, servings/wk							*P*-trend	Every 3.5 servings/wk change^2^

	Decrease			Increase					
≥2	1–1.99^1^	0.5–0.99	No change (±0.49)	0.5–0.99	1–1.99^1^	≥2

Frailty									
Tea									
Events/py	2372/165,775	208/14,644	345/21,999	5827/437,317	294/23,527	171/13,673	2152/171,293		
Model 1	1.08 (1.02, 1.14)	0.95 (0.83, 1.09)	1.00 (0.90, 1.12)	Ref.	0.96 (0.85, 1.08)	0.86 (0.74, 1.00)	0.99 (0.94, 1.04)	0.004	0.97 (0.94, 0.99)
Model 2	1.07 (1.01, 1.13)	1.03 (0.90, 1.18)	1.00 (0.90, 1.11)	Ref.	0.97 (0.86, 1.09)	0.93 (0.79, 1.08)	1.01 (0.96, 1.06)	0.222	0.99 (0.96, 1.01)
Red wine									
Events/py		415/36,303	135/9320	10,339/743,741	115/10,526	365/48,338			
Model 1		1.03 (0.91, 1.16)	1.06 (0.89, 1.25)	Ref.	0.85 (0.71, 1.02)	0.58 (0.52, 0.65)		<0.001	0.75 (0.70, 0.79)
Model 2		1.06 (0.93, 1.20)	1.20 (1.01, 1.42)	Ref.	1.04 (0.87, 1.26)	0.83 (0.74, 0.93)		0.010	0.92 (0.86, 0.98)
Blueberry									
Events/py		448/26,917	253/17,692	9517/722,583	381/26,339	770/54,697			
Model 1		1.60 (1.40, 1.82)	1.17 (1.03, 1.33)	Ref.	0.98 (0.89, 1.09)	0.85 (0.79, 0.91)		<0.001	0.74 (0.69, 0.79)
Model 2		1.31 (1.14, 1.49)	1.11 (0.98, 1.26)	Ref.	1.04 (0.94, 1.15)	0.95 (0.88, 1.02)		<0.001	0.87 (0.81, 0.94)
Apple									
Events/py	2034/159,577	77/8319	271/13,435	7187/516,460	155/9573	59/7688	1586/133,178		
Model 1	1.27 (1.19, 1.34)	1.27 (1.00, 1.61)	1.27 (1.12, 1.43)	Ref.	0.99 (0.84, 1.16)	0.96 (0.74, 1.25)	0.95 (0.90, 1.01)	<0.001	0.83 (0.80, 0.87)
Model 2	1.16 (1.09, 1.23)	1.17 (0.92, 1.47)	1.15 (1.02, 1.30)	Ref.	0.96 (0.81, 1.12)	0.97 (0.75, 1.26)	1.00 (0.94, 1.05)	<0.001	0.91 (0.87, 0.95)
Strawberry									
Events/py		797/59,646	349/21,265	8966/677,041	320/19,025	937/71,250			
Model 1		1.20 (1.09, 1.33)	1.17 (1.05, 1.30)	Ref.	1.13 (1.01, 1.27)	0.99 (0.92, 1.05)		<0.001	0.87 (0.82, 0.93)
Model 2		1.09 (0.98, 1.20)	1.10 (0.99, 1.23)	Ref.	1.07 (0.95, 1.19)	1.02 (0.95, 1.09)		0.070	0.94 (0.88, 1.00)
Orange									
Events/py	1480/120,595	43/3745	362/22,032	7970/578,945	236/15,115	36/3537	1242/104,259		
Model 1	1.14 (1.06, 1.23)	1.24 (0.90, 1.69)	1.10 (0.99, 1.23)	Ref.	1.00 (0.88, 1.14)	1.02 (0.73, 1.42)	0.94 (0.89, 1.00)	<0.001	0.89 (0.85, 0.93)
Model 2	1.08 (1.01, 1.16)	1.21 (0.88, 1.65)	1.05 (0.95, 1.17)	Ref.	0.97 (0.86, 1.11)	1.10 (0.79, 1.53)	0.96 (0.90, 1.02)	0.004	0.94 (0.90, 0.98)
Grapefruit and grapefruit juice									
Events/py	1467/111,019	38/2431	399/29,872	8463/631,194	229/15,163	22/1924	751/56,625		
Model 1	0.93 (0.88, 0.98)	1.03 (0.75, 1.42)	1.00 (0.91, 1.11)	Ref.	1.12 (0.98, 1.28)	0.81 (0.53, 1.23)	0.96 (0.89, 1.03)	0.414	1.02 (0.98, 1.06)
Model 2	0.99 (0.93, 1.04)	1.08 (0.78, 1.48)	0.99 (0.89, 1.09)	Ref.	1.12 (0.99, 1.28)	0.85 (0.56, 1.29)	0.98 (0.91, 1.06)	0.898	1.00 (0.96, 1.04)
Impaired physical function									
Tea									
Events/py	4489/69,869	404/6381	598/9262	11,440/192,197	635/10,330	397/5965	4456/75,135		
Model 1	1.07 (1.03, 1.11)	1.00 (0.91, 1.11)	1.03 (0.95, 1.12)	Ref.	1.01 (0.93, 1.09)	1.06 (0.96, 1.17)	1.03 (1.00, 1.07)	0.204	0.99 (0.97, 1.01)
Model 2	1.05 (1.00, 1.08)	1.00 (0.90, 1.10)	1.02 (0.94, 1.11)	Ref.	1.00 (0.92, 1.08)	1.09 (0.98, 1.20)	1.03 (0.99, 1.07)	0.687	1.00 (0.98, 1.01)
Red wine									
Events/py		994/17,647	260/4174	19,526/316,444	291/5275	1348/25,598			
Model 1		1.02 (0.95, 1.10)	1.07 (0.94, 1.21)	Ref.	0.87 (0.77, 0.97)	0.83 (0.78, 0.87)		<0.001	0.88 (0.85, 0.91)
Model 2		1.01 (0.93, 1.09)	1.06 (0.93, 1.20)	Ref.	0.89 (0.79, 1.00)	0.92 (0.87, 0.98)		0.002	0.94 (0.91, 0.98)
Blueberry									
Events/py		658/11,907	474/7518	19,206/314,540	678/11,391	1403/23,782			
Model 1		1.18 (1.07, 1.32)	1.10 (1.00, 1.20)	Ref.	0.90 (0.83, 0.97)	0.93 (0.88, 0.98)		<0.001	0.89 (0.84, 0.94)
Model 2		1.09 (0.98, 1.21)	1.07 (0.98, 1.17)	Ref.	0.92 (0.85, 0.99)	0.98 (0.93, 1.04)		0.188	0.97 (0.92, 1.02)
Apple									
Events/py	4388/71,514	203/4388	379/4792	13,662/219,582	258/3803	181/4072	3348/60,986		
Model 1	1.10 (1.05, 1.14)	0.92 (0.80, 1.06)	1.16 (1.05, 1.28)	Ref.	1.11 (0.98, 1.26)	0.91 (0.79, 1.06)	0.95 (0.92, 0.99)	<0.001	0.90 (0.87, 0.92)
Model 2	1.04 (1.00, 1.08)	0.90 (0.77, 1.03)	1.09 (0.98, 1.21)	Ref.	1.09 (0.96, 1.23)	0.96 (0.82, 1.11)	0.97 (0.93, 1.01)	<0.001	0.95 (0.92, 0.97)
Strawberry									
Events/py		1551/26,899	585/8670	17,921/293,519	509/7722	1853/32,229			
Model 1		1.08 (1.00, 1.15)	1.05 (0.97, 1.14)	Ref.	1.03 (0.95, 1.13)	0.97 (0.92, 1.01)		<0.001	0.92 (0.88, 0.96)
Model 2		1.04 (0.97, 1.11)	1.01 (0.93, 1.10)	Ref.	1.01 (0.93, 1.10)	0.99 (0.94, 1.04)		0.060	0.96 (0.92, 1.00)
Orange									
Events/py	3269/55,062	91/1881	617/8571	15,203/24,7062	432/6086	99/1872	2708/48,603		
Model 1	1.09 (1.04, 1.14)	1.00 (0.81, 1.24)	1.13 (1.04, 1.23)	Ref.	1.09 (0.99, 1.20)	0.97 (0.79, 1.19)	0.95 (0.91, 0.99)	<0.001	0.92 (0.89, 0.95)
Model 2	1.05 (1.00, 1.10)	1.01 (0.81, 1.24)	1.09 (1.01, 1.18)	Ref.	1.07 (0.97, 1.17)	0.95 (0.77, 1.16)	0.97 (0.93, 1.01)	0.001	0.95 (0.92, 0.98)
Grapefruit and grapefruit juice									
Events/py	3055/49,538	68/1071	798/13,081	16,540/272,594	387/6041	56/1072	1515/25,742		
Model 1	1.00 (0.96, 1.04)	1.06 (0.83, 1.35)	0.99 (0.92, 1.06)	Ref.	1.06 (0.95, 1.17)	0.87 (0.67, 1.14)	0.97 (0.92, 1.02)	0.892	1.00 (0.97, 1.03)
Model 2	1.02 (0.98, 1.06)	1.09 (0.86, 1.39)	0.98 (0.91, 1.05)	Ref.	1.04 (0.94, 1.15)	0.91 (0.70, 1.19)	0.97 (0.91, 1.02)	0.238	0.98 (0.95, 1.01)
Poor mental health									
Tea									
Events/py	1826/128,943	147/11,566	211/17,124	4764/333,198	207/18,225	125/10,728	1664/132,896		
Model 1	1.01 (0.95, 1.07)	0.87 (0.74, 1.02)	0.84 (0.73, 0.96)	Ref.	0.78 (0.68, 0.89)	0.79 (0.66, 0.94)	0.90 (0.85, 0.95)	0.016	0.97 (0.94, 0.99)
Model 2	1.05 (0.99, 1.11)	0.94 (0.80, 1.11)	0.88 (0.77, 1.01)	Ref.	0.83 (0.72, 0.95)	0.85 (0.71, 1.02)	0.95 (0.90, 1.00)	0.074	0.98 (0.95, 1.00)
Red wine									
Events/py		327/27,757	85/7330	8019/572,166	92/8182	421/37,245			
Model 1		0.96 (0.84, 1.09)	0.88 (0.71, 1.09)	Ref.	0.80 (0.65, 0.99)	0.85 (0.77, 0.94)		0.035	0.94 (0.88, 1.00)
Model 2		1.02 (0.89, 1.17)	0.94 (0.76, 1.17)	Ref.	0.95 (0.77, 1.17)	1.04 (0.93, 1.16)		0.472	1.02 (0.96, 1.09)
Blueberry									
Events/py		234/21,032	177/13,801	7823/555,947	255/20,406	455/41,495			
Model 1		1.23 (1.03, 1.47)	0.99 (0.85, 1.15)	Ref.	0.81 (0.72, 0.92)	0.86 (0.78, 0.95)		<0.001	0.83 (0.76, 0.91)
Model 2		1.14 (0.95, 1.35)	0.98 (0.84, 1.14)	Ref.	0.85 (0.75, 0.97)	0.94 (0.85, 1.03)		0.070	0.92 (0.84, 1.01)
Apple									
Events/py	1661/124,956	69/6707	201/10,499	5635/394,087	110/7267	58/6106	1210/103,059		
Model 1	0.99 (0.93, 1.06)	0.89 (0.70, 1.14)	1.14 (0.99, 1.31)	Ref.	1.08 (0.89, 1.30)	0.84 (0.65, 1.09)	0.90 (0.85, 0.96)	0.001	0.93 (0.89, 0.97)
Model 2	1.00 (0.93, 1.07)	0.91 (0.71, 1.16)	1.12 (0.97, 1.29)	Ref.	1.05 (0.87, 1.27)	0.88 (0.68, 1.15)	0.96 (0.90, 1.02)	0.265	0.98 (0.93, 1.02)
Strawberry									
Events/py		545/46,645	284/16,289	7282/520,395	228/14,539	605/54,812			
Model 1		1.00 (0.89, 1.12)	1.19 (1.05, 1.34)	Ref.	1.06 (0.93, 1.21)	0.87 (0.80, 0.94)		<0.001	0.85 (0.79, 0.92)
Model 2		0.99 (0.88, 1.11)	1.18 (1.05, 1.33)	Ref.	1.05 (0.92, 1.20)	0.90 (0.83, 0.98)		0.001	0.88 (0.82, 0.95)
Orange									
Events/py	1296/94,827	30/3125	282/17,007	6143/441,370	173/11,622	34/2838	986/81,892		
Model 1	0.94 (0.87, 1.01)	0.73 (0.51, 1.06)	1.07 (0.95, 1.21)	Ref.	1.09 (0.94, 1.27)	0.93 (0.66, 1.30)	0.95 (0.89, 1.01)	0.056	0.95 (0.91, 1.00)
Model 2	0.96 (0.89, 1.04)	0.77 (0.53, 1.11)	1.09 (0.96, 1.23)	Ref.	1.09 (0.94, 1.27)	0.99 (0.70, 1.39)	1.00 (0.93, 1.07)	0.405	0.98 (0.93, 1.03)
Grapefruit and grapefruit juice									
Events/py	1140/87,711	22/1873	342/22,387	6677/483,204	159/11,708	16/1578	588/44,221		
Model 1	0.91 (0.85, 0.97)	0.81 (0.53, 1.23)	1.09 (0.98, 1.22)	Ref.	0.97 (0.83, 1.13)	0.73 (0.44, 1.19)	0.95 (0.87, 1.04)	0.073	1.04 (1.00, 1.09)
Model 2	0.95 (0.89, 1.01)	0.87 (0.57, 1.32)	1.10 (0.99, 1.23)	Ref.	0.96 (0.82, 1.12)	0.72 (0.44, 1.18)	0.98 (0.90, 1.07)	0.348	1.02 (0.98, 1.07)

Hazard ratios (95% CI) for frailty, physical impairment, and poor mental health during 24 y of follow-up, obtained from Cox proportional hazards models. Model 1 adjusted for baseline age, questionnaire cycle, and intakes of the exposure variable of interest; model 2 adjusted for baseline age; questionnaire cycle; ethnicity; smoking status; change in smoking status; marital status; menopausal status; a family history of myocardial infarction, diabetes, and cancer; multivitamin use; use of aspirin; use of other medications; a history of hypertension, hypercholesterolemia, diabetes, myocardial infarction, and stroke; physical activity; change in physical activity; BMI; change in BMI; intakes of the exposure variable of interest; and both intakes and change in intakes of alcohol, total energy, meat, nuts, saturated fat, polyunsaturated fat, trans fat, cereal fiber, and soft drink. Exposures with blocked cells were categorized into only 5 change categories due to their narrower distribution.

Abbreviation: py, person years.

1 Except for tea, where the hazard ratio (95% CI) is presented for a 4-y change in intake of 1 serve/d.

2 Except for red wine and strawberries where this is ≥1.

Among participants of the HPFS, compared with flavodiet scores that did not change, scores that decreased by ≥7 servings/wk were associated with 60% higher risk of poor mental health (HR_Q5vsQ1_: 1.60; 95% CI: 1.32, 1.95) and a 3-servings/d increase in flavodiet score was associated with 15% lower risk of poor mental health (HR_Q5vsQ1_: 0.85; 95% CI: 0.72, 1.00), after multivariable adjustments (model 2, Supplemental Table 4). Participants with the greatest decreases in apple intake had 20% higher risk of poor mental health, an increase in grapefruit intake by 1 or more serves per day was associated with 28%–34% higher risk of impaired physical function, and a 1-serve/d increase in tea intake was associated with 8% lower risk of poor mental health (model 2, Supplemental Table 4).

### Sensitivity analyses

When death was included as an event, associations between both total flavonoid and flavodiet score intakes and each of the 3 outcomes were not meaningfully changed in the NHS, although in the HPFS cohort, associations were attenuated as HRs increased (Supplemental Table 5). When follow-up time was truncated to 12 y in the NHS, associations between both flavodiet score and total flavonoid intakes and each of the 3 outcomes were weaker and less stable (Supplemental Table 6). Left-truncating the data so that NHS participants entered the study only at 70 y of age did not materially alter the observed associations between the flavodiet score or total flavonoid intake and each of the 3 outcomes (Supplemental Table 7).

## Discussion

Among females in the NHS, both habitually high flavodiet scores and increases in flavodiet scores were associated with modestly lower risk of frailty, impaired physical function, and poor mental health. For the individual flavonoid-rich foods, higher habitual intakes and increases in intakes of tea, red wine, apples, blueberries, and oranges/orange juice tended to be associated with lower risk of all outcomes. Furthermore, higher intakes of total flavonoids and all flavonoid subclasses were associated with lower risk of most outcomes. Unexpectedly, fewer associations were observed in males from HPFS, indicating that further work will be needed to better understand if there may be sex differences in dietary risk factors for health in aging.

The significance of diet—both quantity and quality—in mitigating frailty is widely acknowledged, with higher protein intake [23], better diet quality [19], and a higher consumption of healthy plant-based foods [24] consistently being identified as key factors. Dietary antioxidant capacity has been linked to a lower frailty risk [23], with a recent Framingham Heart Study finding that only higher flavonol intake reduced frailty odds [25]. In this study, flavodiet score, total flavonoids, and all flavonoid subclasses were associated with lower risk of frailty among females in the NHS. Among individual flavonoid-rich foods, habitually high intakes, and half-serving per day increases in intakes, of blueberries, apples, and oranges/orange juice were each linked to a lower frailty risk, aligning with previously evidence that fruit intake is inversely associated with frailty [26]. In Asian populations, regular tea consumption is associated with lower risk of frailty [[27], [28], [29]]. In this study, participants with moderate intakes of red wine had lowest risk of frailty compared with nonconsumers in line with findings that a moderate alcohol intake with ≥80% of alcohol coming from wine, and drinking only with meals is associated with lower risk of frailty [30]. Collectively, the evidence suggests that increasing the consumption of flavonoid-rich foods and beverages, such as tea, red wine, and fruits, during midlife may play a preventive role in delaying the onset of frailty among females.

Acknowledging the significance of physical function in maintaining independence and overall well-being among aging individuals, our results align with previous research indicating that individuals with healthier diets, as assessed by the Alternative Healthy Eating Index-2010, exhibit lower risk of impaired physical function [20,31]. In randomized controlled trials, flavonoids have been shown to increase skeletal muscle mass and gait speed in middle aged and elderly participants with and without sarcopenia [32]. Furthermore, ≤12 wk of flavonoid-rich cocoa intervention has been shown to improve physical performance and mobility with reductions noted in oxidative stress and inflammation biomarkers thought to be partly accounting for the observed effects [33]. In this study, an increase in the flavodiet score was linked to lower risk of impaired physical function and habitually high intakes of red wine, blueberries, apples, and oranges/orange juice and half-serving per day increases of red wine, apples and oranges/orange juice were linked to lower risk of impaired physical function among females in the NHS. Notably, previous evidence demonstrating the positive influence of red wine polyphenols on aging-related declines in physical exercise in rats aligns with our findings [34], emphasizing the potential of flavonoids to mitigate impairments in physical function among aging individuals.

This study contributes to the growing body of evidence supporting a potential link between flavonoids and a reduction in depression symptoms [35,36]. Our findings align with previous research in the NHS and NHS II, where females with the highest intakes of flavonols, flavones, and flavanones had 7%–10% lower risk of depression than those with the lowest intakes [33]. Among males in the HPFS, moderate to high intakes of flavonols, flavan-3-ol polymers, anthocyanins, and flavones were associated with lower risk of poor mental health although associations were not as pronounced as for the NHS. Furthermore, a habitually higher, and increase in, flavodiet score was linked to lower risk of poor mental health in the HPFS. Our findings among NHS participants that consistently high intakes of apples, strawberries, oranges/orange juice, and grapefruit/grapefruit juice were linked to lower risk of poor mental health echo existing research pointing a benefit of fruit intake for mental health [37,38] and further suggest that this may be attributed to the high flavonoid content of several fruits. In the HPFS, moderate intakes of red wine, habitually high intakes of tea and blueberries, and increases in intakes of tea, were linked to lower risk of poor mental health. The observed association between wine consumption and a lower incidence of poor mental health is consistent with literature suggesting that moderate wine consumption may reduce depression incidence, although heavy drinking could elevate risk [39]. Previous research reports associations between higher tea intake with a lower risk of depression and indicating a multifaceted impact of tea flavonoids, and their metabolites, in collectively reducing depression risk through multiple pathways [40]. Additionally, reduced risk of poor mental health associated with higher blueberry intakes contributes to the expanding evidence suggesting cognitive and mood benefits linked to blueberries [41].

It is important to note that although less consistent associations were found among males in the HPFS, this should not discount the potential impact of flavonoids on aging outcomes within this demographic. Notably, sex-specific associations have not been extensively reported in existing literature; the majority of previous studies were conducted in cohorts consisting solely of either males or females or failed to examine sex-specific differences. The lack of associations in males in this study might be attributed to the comparatively shorter follow-up time resulting in fewer events and reduced statistical power. Supporting this, truncating follow-up time to 12 y in the NHS resulted in weaker and less stable associations. Additionally, the lower prevalence of current smokers among males, in contrast to females, is noteworthy, given evidence indicating that associations between flavonoids and various health outcomes are more pronounced in smokers than nonsmokers [42]. This is supported by the observation that, in Asian populations—where smoking is more prevalent among males than females [43]—tea intake was associated with lower risk of frailty only among males [[27], [28], [29]]. More research into whether these associations are truly sex specific is warranted.

The limitations of our study also warrant discussion. Our outcomes were derived from self-reported questionnaire data, which may result in outcome misclassification, although the direction of any potential bias is uncertain. Although we adjusted for possible confounders that are strongly associated with risk of frailty, physical function, and mental health, these were mostly self-reported and there is still the possibility of residual or unmeasured confounding from additional unmeasured factors. It is possible that our findings might be due to other constituents found in the foods that contribute most to flavonoid intake; however, in a population-based study like ours, it is impossible to disentangle the relative influence of all constituents of flavonoid-rich foods. Given the number of statistical tests performed in this analysis, readers may wish to consider using a more conservative significance threshold (e.g., *P* < 0.001, derived from 0.05/50 using the Bonferroni correction) to account for multiple comparisons. This study only focused on middle-aged and older females and males who were predominantly White and further studies are needed to examine associations in diverse population groups and in populations with broader intakes of these flavonoid-rich foods.

By mitigating risks of frailty, impaired physical function, and poor mental health, habitual consumption of key flavonoid-rich foods—such as blueberries, apples, red wine, oranges, and tea—may augment healthy aging. From a public health standpoint, a modest yet achievable adjustment, involving an increase of 3 servings/d in flavonoid-rich foods, translated to 6%–11% lower risk across all 3 outcomes in females and 15% lower risk of poor mental health in males. Overall, these findings underscore the potential for simple dietary modifications to impact overall quality of life and contribute to the optimization of healthy aging.

## Author contributions

The authors’ responsibilities were as follows – AC, EBR: were responsible for the study concept and design; EBR: collected the data; NPB, AC, YLL, EBR: performed all statistical analyses and interpreted data; NPB, AC: drafted the manuscript; FG, EBR: critically appraised the manuscript; and all authors: read and approved the final manuscript.

## Data availability

Data described in the article, code book, and analytic code will be made available upon request pending approval by the Channing Division of Network Medicine at Brigham and Women’s Hospital and Harvard Medical School. Further information including the procedures to obtain and access data from the Nurses’ Health Study and the Health Professionals Follow-Up Study is described at https://www.nurseshealthstudy.org/researchers (contact e-mail: nhsaccess@channing.harvard.edu) and https://sites.sph.harvard.edu/hpfs/for-collaborators/.

## Declaration of Generative AI and AI-assisted technologies in the writing process

During the preparation of this work the authors used ChatGPT for English language optimization. After using this tool, the authors reviewed and edited the content as needed and take full responsibility for the content of the publication.

## Funding

This work was supported by grants from the National Institutes of Health (UM1 CA186107 and U01CA167552) and by funding from The US Highbush Blueberry Council (USHBC) with oversight from the USDA. Funders had no role in study design; collection, analysis, and interpretation of data; writing of manuscript; nor any restrictions regarding publication.

## Conflicts of interest

AC and EBR act as advisors to the USHBC grant committee. All other authors report no conflicts of interest.
