# Supplementary material for: Associations between flavonoid-rich food and flavonoid intakes and incident unhealthy aging outcomes in older United States males and females
Source: Am J Clin Nutr. 2025 Feb 15;121(5):972–85. doi: 10.1016/j.ajcnut.2025.02.010 (PMC12107487; doi:10.1016/j.ajcnut.2025.02.010)
Supplement: Multimedia component 1 [file mmc1.docx]

**Associations between flavonoid-rich food and flavonoid intakes and incident unhealthy aging outcomes in older U.S. males and females**

Supplementary Material

*Bondonno et al.*

| **Supplementary Table 1.** Associations between time-updated flavonoid-rich food intakes and healthy aging domains in the Health Professionals Follow-up Study | | | | | |
| --- | --- | --- | --- | --- | --- |
|  | Quartiles of intake | | | |  |
|  | Q1 | Q2 | Q3 | Q4 | p-trend |
| Frailty |  |  |  |  |  |
| Tea |  |  |  |  |  |
| Intake (s/w) | 0 [0 – 0] | 0.5 [0.5 – 0.5] | 1.5 [1.0 – 3.0] | 7.0 [6.0 – 17.5] |  |
| Events/py | 847/79672 | 224/22987 | 432/45326 | 454/50387 |  |
| Model 1 | Ref. | 0.91 (0.78, 1.06) | 0.99 (0.88, 1.11) | 0.96 (0.86, 1.08) | 0.420 |
| Model 2 | Ref. | 0.92 (0.79, 1.07) | 1.02 (0.90, 1.15) | 1.03 (0.91, 1.16) | 0.137 |
| Red wine |  |  |  |  |  |
| Intake (s/w) | 0 [0 – 0] | 0.5 [0.5 – 0.5] | 3.0 [1.0 – 3.0] | 7.0 [5.5 – 7.0] |  |
| Events/py | 1010/80426 | 353/36014 | 396/54418 | 198/27514 |  |
| Model 1 | Ref. | 0.86 (0.76, 0.97) | 0.71 (0.63, 0.80) | 0.71 (0.61, 0.83) | 0.001 |
| Model 2 | Ref. | 0.95 (0.82, 1.10) | 0.89 (0.76, 1.03) | 0.92 (0.76, 1.12) | 0.844 |
| Blueberry | | |  |  |  |
| Intake (s/w) | 0 [0 – 0] | 0.5 [0.5 – 0.5] | 1.0 [1.0 – 1.0] | 3.0 [3.0 – 5.5] |  |
| Events/py | 691/66321 | 555/59853 | 298/30073 | 413/42124 |  |
| Model 1 | Ref. | 0.93 (0.83, 1.04) | 0.91 (0.79, 1.05) | 0.76 (0.67, 0.86) | 0.049 |
| Model 2 | Ref. | 1.04 (0.92, 1.17) | 1.07 (0.93, 1.24) | 0.98 (0.85, 1.12) | 0.404 |
| Apple |  |  |  |  |  |
| Intake (s/w) | 0.5 [0 – 0.5] | 1.0 [1.0 – 1.0] | 3.0 [3.0 – 3.0] | 7.0 [5.5 – 7.0] |  |
| Events/py | 822/73238 | 401/40484 | 503/54740 | 231/29910 |  |
| Model 1 | Ref. | 0.92 (0.81, 1.04) | 0.85 (0.76, 0.95) | 0.75 (0.65, 0.87) | <0.001 |
| Model 2 | Ref. | 1.02 (0.90, 1.16) | 0.99 (0.88, 1.12) | 0.96 (0.82, 1.12) | 0.471 |
| Strawberry | | |  |  |  |
| Intake (s/w) | 0 [0 – 0] | 0.5 [0.5 – 0.5] | 1.0 [1.0 – 1.0] | 3.0 [3.0 – 3.0] |  |
| Events/py | 558/49183 | 682/75207 | 375/39951 | 342/34032 |  |
| Model 1 | Ref. | 0.83 (0.74, 0.94) | 0.82 (0.72, 0.94) | 0.73 (0.63, 0.83) | 0.023 |
| Model 2 | Ref. | 0.90 (0.80, 1.01) | 0.92 (0.80, 1.06) | 0.85 (0.73, 0.98) | 0.653 |
| Orange | |  |  |  |  |
| Intake (s/w) | 0 [0 – 0] | 0.5 [0.5 – 0.5] | 1.0 [1.0 – 1.0] | 3.0 [3.0 – 5.5] |  |
| Events/py | 551/48792 | 489/56891 | 353/36729 | 564/55960 |  |
| Model 1 | Ref. | 0.85 (0.75, 0.96) | 0.91 (0.8, 1.05) | 0.94 (0.83, 1.06) | 0.692 |
| Model 2 | Ref. | 0.89 (0.78, 1.01) | 1.00 (0.87, 1.15) | 1.07 (0.94, 1.21) | 0.310 |
| Grapefruit and grapefruit juice | | |  |  |  |
| Intake (s/w) | 0 [0 – 0] | 0.3 [0.3 – 0.3] | 0.7 [0.7 – 0.7] | 2.0 [2.0 – 4.7] |  |
| Events/py | 1358/127270 | 250/34664 | 154/16597 | 195/19841 |  |
| Model 1 | Ref. | 0.86 (0.75, 0.98) | 0.84 (0.71, 1.00) | 0.92 (0.79, 1.08) | 0.392 |
| Model 2 | Ref. | 0.94 (0.82, 1.08) | 0.88 (0.73, 1.04) | 1.06 (0.90, 1.24) | 0.788 |
| Physical function | |  |  |  |  |
| Tea |  |  |  |  |  |
| Intake (s/w) | 0 [0 – 0] | 0.5 [0.5 – 0.5] | 2.0 [1.0 – 3.0] | 7.5 [6.5 – 17.5] |  |
| Events/py | 1784/54000 | 501/15720 | 955/33300 | 925/33699 |  |
| Model 1 | Ref. | 0.97 (0.87, 1.07) | 0.97 (0.89, 1.05) | 0.93 (0.86, 1.01) | 0.449 |
| Model 2 | Ref. | 0.98 (0.89, 1.09) | 0.98 (0.90, 1.06) | 0.98 (0.91, 1.07) | 0.721 |
| Red wine |  |  |  |  |  |
| Intake (s/w) | 0 [0 – 0] | 0.5 [0.5 – 0.5] | 3.0 [1.0 – 3.0] | 7.0 [5.5 – 7.0] |  |
| Events/py | 1833/51905 | 763/24500 | 1036/39865 | 533/20451 |  |
| Model 1 | Ref. | 0.95 (0.87, 1.03) | 0.84 (0.77, 0.90) | 0.80 (0.72, 0.88) | 0.001 |
| Model 2 | Ref. | 0.98 (0.89, 1.09) | 0.91 (0.82, 1.01) | 0.89 (0.78, 1.01) | 0.177 |
| Blueberry | | |  |  |  |
| Intake (mg/d) | 0 [0 – 0] | 0.5 [0.5 – 0.5] | 1.0 [1.0 – 1.0] | 3.0 [3.0 – 5.5] |  |
| Events/py | 1293/43054 | 1216/41831 | 633/21498 | 1023/30337 |  |
| Model 1 | Ref. | 0.97 (0.89, 1.05) | 0.88 (0.80, 0.97) | 0.83 (0.76, 0.90) | 0.002 |
| Model 2 | Ref. | 1.03 (0.95, 1.12) | 0.97 (0.88, 1.07) | 1.00 (0.91, 1.09) | 0.779 |
| Apple |  |  |  |  |  |
| Intake (s/w) | 0.5 [0 – 0.5] | 1.0 [1.0 – 1.0] | 3.0 [3.0 – 3.0] | 7.0 [5.5 – 7.0] |  |
| Events/py | 1595/48858 | 880/28234 | 1131/38149 | 559/21478 |  |
| Model 1 | Ref. | 0.97 (0.89, 1.05) | 0.90 (0.83, 0.97) | 0.83 (0.75, 0.92) | 0.002 |
| Model 2 | Ref. | 1.03 (0.94, 1.12) | 1.00 (0.92, 1.08) | 1.01 (0.91, 1.12) | 0.500 |
| Strawberry | | |  |  |  |
| Intake (s/w) | 0 [0 – 0] | 0.5 [0.5 – 0.5] | 1.0 [1.0 – 1.0] | 3.0 [3.0 – 3.0] |  |
| Events/py | 1042/32565 | 1503/52036 | 868/28422 | 752/23696 |  |
| Model 1 | Ref. | 0.93 (0.86, 1.01) | 0.93 (0.85, 1.02) | 0.81 (0.74, 0.90) | 0.001 |
| Model 2 | Ref. | 0.96 (0.88, 1.04) | 0.98 (0.89, 1.08) | 0.92 (0.83, 1.02) | 0.320 |
| Orange | | |  |  |  |
| Intake (s/w) | 0 [0 – 0] | 0.5 [0.5 – 0.5] | 1.0 [1.0 – 1.0] | 3.0 [3.0 – 5.5] |  |
| Events/py | 1135/33082 | 1178/39973 | 741/25660 | 1111/38005 |  |
| Model 1 | Ref. | 0.97 (0.89, 1.05) | 0.95 (0.86, 1.04) | 0.95 (0.87, 1.04) | 0.416 |
| Model 2 | Ref. | 1.00 (0.92, 1.09) | 1.02 (0.92, 1.12) | 1.07 (0.97, 1.17) | 0.137 |
| Grapefruit and grapefruit juice | | |  |  |  |
| Intake (s/w) | 0 [0 – 0] | 0.3 [0.3 – 0.3] | 0.7 [0.7 – 0.7] | 2.0 [2.0 – 3.7] |  |
| Events/py | 2891/86565 | 576/25205 | 329/11348 | 369/13601 |  |
| Model 1 | Ref. | 0.86 (0.79, 0.95) | 0.91 (0.80, 1.02) | 0.85 (0.76, 0.95) | 0.007 |
| Model 2 | Ref. | 0.91 (0.83, 1.00) | 0.96 (0.85, 1.08) | 0.97 (0.86, 1.08) | 0.436 |
| Mental health | |  |  |  |  |
| Tea |  |  |  |  |  |
| Intake (s/w) | 0 [0 – 0] | 0.5 [0.5 – 0.5] | 1.5 [1.0 – 3.0] | 7.0 [6.0 – 17.5] |  |
| Events/py | 742/79845 | 196/23041 | 365/45467 | 366/50511 |  |
| Model 1 | Ref. | 0.91 (0.78, 1.07) | 0.92 (0.81, 1.04) | 0.84 (0.74, 0.96) | 0.099 |
| Model 2 | Ref. | 0.94 (0.80, 1.10) | 0.94 (0.82, 1.07) | 0.86 (0.76, 0.98) | 0.162 |
| Red wine |  |  |  |  |  |
| Intake (s/w) | 0 [0 – 0] | 0.5 [0.5 – 0.5] | 3.0 [1.0 – 3.0] | 7.0 [5.5 – 7.0] |  |
| Events/py | 844/81223 | 300/36034 | 342/54363 | 183/27244 |  |
| Model 1 | Ref. | 0.88 (0.77, 1.00) | 0.71 (0.62, 0.80) | 0.74 (0.63, 0.87) | 0.058 |
| Model 2 | Ref. | 0.92 (0.79, 1.07) | 0.78 (0.66, 0.91) | 0.82 (0.67, 1.00) | 0.967 |
| Blueberry | | |  |  |  |
| Intake (s/w) | 0 [0 – 0] | 0.5 [0.5 – 0.5] | 1.0 [1.0 – 1.0] | 3.0 [3.0 – 5.5] |  |
| Events/py | 612/66678 | 457/59901 | 238/30198 | 362/42086 |  |
| Model 1 | Ref. | 0.83 (0.73, 0.94) | 0.79 (0.68, 0.92) | 0.75 (0.66, 0.86) | 0.002 |
| Model 2 | Ref. | 0.89 (0.78, 1.01) | 0.88 (0.75, 1.02) | 0.85 (0.74, 0.98) | 0.073 |
| Apple |  |  |  |  |  |
| Intake (s/w) | 0.5 [0 – 0.5] | 1.0 [1.0 – 1.0] | 3.0 [3.0 – 3.0] | 7.0 [5.5 – 7.0] |  |
| Events/py | 703/73322 | 341/40689 | 404/54951 | 221/29901 |  |
| Model 1 | Ref. | 0.92 (0.80, 1.05) | 0.81 (0.71, 0.91) | 0.86 (0.73, 1.00) | 0.032 |
| Model 2 | Ref. | 0.98 (0.86, 1.12) | 0.89 (0.78, 1.01) | 0.99 (0.84, 1.16) | 0.635 |
| Strawberry | | |  |  |  |
| Intake (s/w) | 0 [0 – 0] | 0.5 [0.5 – 0.5] | 1.0 [1.0 – 1.0] | 3.0 [3.0 – 3.0] |  |
| Events/py | 466/48894 | 588/75611 | 328/40035 | 287/34324 |  |
| Model 1 | Ref. | 0.86 (0.76, 0.97) | 0.90 (0.78, 1.04) | 0.78 (0.67, 0.91) | 0.016 |
| Model 2 | Ref. | 0.91 (0.80, 1.03) | 0.98 (0.85, 1.14) | 0.86 (0.74, 1.01) | 0.133 |
| Orange | | |  |  |  |
| Intake (s/w) | 0 [0 – 0] | 0.5 [0.5 – 0.5] | 1.0 [1.0 – 1.0] | 3.0 [3.0 – 5.5] |  |
| Events/py | 487/48918 | 425/56809 | 298/36725 | 459/56412 |  |
| Model 1 | Ref. | 0.80 (0.70, 0.91) | 0.87 (0.75, 1.01) | 0.85 (0.74, 0.96) | 0.470 |
| Model 2 | Ref. | 0.84 (0.74, 0.96) | 0.93 (0.80, 1.08) | 0.92 (0.80, 1.06) | 0.930 |
| Grapefruit and grapefruit juice | | |  |  |  |
| Intake (s/w) | 0 [0 – 0] | 0.3 [0.3 – 0.3] | 0.7 [0.7 – 0.7] | 2.0 [2.0 – 4.7] |  |
| Events/py | 1158/127594 | 214/34535 | 133/16729 | 164/20005 |  |
| Model 1 | Ref. | 0.81 (0.70, 0.94) | 0.88 (0.73, 1.06) | 0.94 (0.80, 1.11) | 0.675 |
| Model 2 | Ref. | 0.86 (0.74, 1.00) | 0.91 (0.75, 1.09) | 1.02 (0.86, 1.21) | 0.790 |
| Hazard ratios (95% CI) for frailty, physical impairment and poor mental health during 12 years of follow up, obtained from Cox proportional hazards models. Model 1 adjusted for age and questionnaire cycle; Model 2 adjusted for age, questionnaire cycle, ethnicity, smoking status, marital status, family history of myocardial infarction, diabetes and cancer, multivitamin use, use of aspirin, use of other medications, history of hypertension, hypercholesterolemia, diabetes, myocardial infarction, and stroke, physical activity, BMI, and intakes of alcohol, total energy, meat, nuts, saturated fat, polyunsaturated fat, trans fat, cereal fibre, and soft drink. Intakes (serves per week) are reported as median [p25 – p75]. Py, person years. | | | | | |

| **Supplementary Table 2**. Associations between time-updated flavonoid subclass intakes and healthy aging domains in the Nurses’ Health Study I | | | | | | |
| --- | --- | --- | --- | --- | --- | --- |
|  | Quintiles of flavonoid intake | | | | | p-trend |
|  | Q1 | Q2 | Q3 | Q4 | Q5 |  |
| Frailty |  |  |  |  |  |  |
| Flavonols |  |  |  |  |  |  |
| Intake (mg/d) | 6.7 [5.3 – 7.7] | 10.7 [9.8 – 11.7] | 15.0 [13.8 – 16.2] | 21.5 [19.4 – 24.3] | 36.8 [32.0 – 46.1] |  |
| Events/py | 2849/152904 | 2567/167244 | 2185/175387 | 1973/177325 | 1795/175370 |  |
| Model 1 | Ref. | 0.87 (0.82, 0.91) | 0.74 (0.70, 0.78) | 0.69 (0.65, 0.73) | 0.66 (0.62, 0.70) | <0.001 |
| Model 2 | Ref. | 0.97 (0.92, 1.03) | 0.89 (0.84, 0.95) | 0.86 (0.81, 0.92) | 0.83 (0.78, 0.89) | <0.001 |
| Flavan-3-ols monomers | | |  |  |  |  |
| Intake (mg/d) | 7.1 [4.6 – 8.9] | 14.1 [12.4 – 15.9] | 23.7 [20.5 – 29.8] | 62.5 [44.3 – 73.3] | 174.3 [89.8 – 184.1] |  |
| Events/py | 2791/154580 | 2410/169088 | 2156/175246 | 1958/175970 | 2054/173416 |  |
| Model 1 | Ref. | 0.81 (0.77, 0.85) | 0.72 (0.68, 0.76) | 0.66 (0.62, 0.70) | 0.70 (0.66, 0.74) | <0.001 |
| Model 2 | Ref. | 0.93 (0.88, 0.98) | 0.92 (0.86, 0.97) | 0.86 (0.81, 0.92) | 0.89 (0.84, 0.95) | 0.006 |
| Flavan-3-ols polymers | | |  |  |  |  |
| Intake (mg/d) | 37.3 [26.0 – 47.5] | 80.2 [69.4 – 91.9] | 136.6 [119.5 – 155.5] | 231.0 [203.3 – 259.3] | 546.9 [380.5 – 629.3] |  |
| Events/py | 2651/154207 | 2434/169005 | 2250/175710 | 2071/175570 | 1963/173738 |  |
| Model 1 | Ref. | 0.86 (0.82, 0.91) | 0.77 (0.73, 0.82) | 0.72 (0.68, 0.77) | 0.70 (0.66, 0.75) | <0.001 |
| Model 2 | Ref. | 0.97 (0.91, 1.02) | 0.93 (0.88, 0.99) | 0.91 (0.85, 0.97) | 0.91 (0.85, 0.97) | 0.007 |
| Anthocyanins |  |  |  |  |  |  |
| Intake (mg/d) | 1.3 [0.6 – 2.1] | 3.8 [3.1 – 4.2] | 6.2 [5.4 – 8.0] | 14.0 [13.2 – 15.1] | 23.1 [21.2 – 28.7] |  |
| Events/py | 2805/149423 | 2597/167444 | 2331/174420 | 1902/176859 | 1734/180082 |  |
| Model 1 | Ref. | 0.84 (0.80, 0.89) | 0.78 (0.74, 0.82) | 0.65 (0.61, 0.69) | 0.59 (0.56, 0.64) | <0.001 |
| Model 2 | Ref. | 0.96 (0.91, 1.01) | 0.96 (0.91, 1.02) | 0.89 (0.84, 0.95) | 0.87 (0.81, 0.93) | <0.001 |
| Flavanones |  |  |  |  |  |  |
| Intake (mg/d) | 4.7 [0.6 – 7.6] | 15.9 [12.6 – 21.6] | 35.4 [30.8 – 43.2] | 57.8 [55.0 – 61.4] | 83.2 [71.7 – 107.8] |  |
| Events/py | 2585/156318 | 2065/160558 | 2238/169678 | 2336/180766 | 2145/180909 |  |
| Model 1 | Ref. | 0.82 (0.77, 0.86) | 0.81 (0.77, 0.86) | 0.74 (0.70, 0.78) | 0.67 (0.63, 0.71) | <0.001 |
| Model 2 | Ref. | 0.92 (0.87, 0.97) | 0.94 (0.88, 0.99) | 0.88 (0.83, 0.93) | 0.84 (0.79, 0.89) | <0.001 |
| Flavones |  |  |  |  |  |  |
| Intake (mg/d) | 0.5 [0.4 – 0.6] | 1.0 [0.9 – 1.2] | 1.7 [1.5 – 1.9] | 2.4 [2.3 – 2.6] | 3.5 [3.1 – 4.2] |  |
| Events/py | 2745/152346 | 2373/164599 | 2295/171232 | 2041/180861 | 1915/179192 |  |
| Model 1 | Ref. | 0.83 (0.79, 0.88) | 0.77 (0.73, 0.81) | 0.67 (0.63, 0.71) | 0.65 (0.61, 0.69) | <0.001 |
| Model 2 | Ref. | 0.93 (0.88, 0.99) | 0.90 (0.85, 0.95) | 0.84 (0.79, 0.89) | 0.84 (0.79, 0.89) | <0.001 |
| Impaired physical function | | |  |  |  |  |
| Flavonols |  |  |  |  |  |  |
| Intake (mg/d) | 6.7 [5.4 – 7.8] | 10.8 [9.8 – 11.7] | 15.0 [13.8 – 16.2] | 21.5 [19.4 – 24.3] | 36.8 [32.1 – 46.2] |  |
| Events/py | 4135/56937 | 4618/69264 | 4677/78354 | 4542/81746 | 4447/82837 |  |
| Model 1 | Ref. | 0.95 (0.91, 0.99) | 0.87 (0.83, 0.90) | 0.83 (0.79, 0.86) | 0.81 (0.78, 0.85) | <0.001 |
| Model 2 | Ref. | 0.99 (0.95, 1.03) | 0.93 (0.89, 0.97) | 0.90 (0.86, 0.95) | 0.90 (0.85, 0.94) | <0.001 |
| Flavan-3-ols monomers | | |  |  |  |  |
| Intake (mg/d) | 7.1 [4.7 – 8.9] | 14.2 [12.4 – 15.9] | 23.7 [20.5 – 29.5] | 62.2 [44.2 – 73.3] | 173.9 [88.4 – 183.7] |  |
| Events/py | 4149/58581 | 4541/71628 | 4652/79355 | 4569/80887 | 4508/78686 |  |
| Model 1 | Ref. | 0.91 (0.87, 0.94) | 0.85 (0.81, 0.88) | 0.82 (0.79, 0.85) | 0.82 (0.79, 0.86) | <0.001 |
| Model 2 | Ref. | 0.96 (0.92, 1.00) | 0.94 (0.90, 0.99) | 0.92 (0.87, 0.96) | 0.92 (0.88, 0.96) | 0.003 |
| Flavan-3-ols polymers | | |  |  |  |  |
| Intake (mg/d) | 37.8 [26.6 – 47.6] | 80.7 [69.7 – 92.5] | 136.3 [119.2 – 155.5] | 231.3 [203.3 – 259.5] | 543.7 [370.6 – 624.2] |  |
| Events/py | 4201/60341 | 4507/71284 | 4681/77669 | 4616/79616 | 4414/80228 |  |
| Model 1 | Ref. | 0.93 (0.89, 0.97) | 0.89 (0.85, 0.92) | 0.85 (0.81, 0.88) | 0.81 (0.78, 0.84) | <0.001 |
| Model 2 | Ref. | 0.98 (0.94, 1.02) | 0.97 (0.93, 1.01) | 0.94 (0.90, 0.98) | 0.91 (0.87, 0.95) | <0.001 |
| Anthocyanins |  |  |  |  |  |  |
| Intake (mg/d) | 1.4 [0.7 – 2.1] | 3.7 [3.1 – 4.2] | 6.2 [5.4 – 8.0] | 14.1 [13.2 – 15.1] | 23.2 [21.2 – 29.0] |  |
| Events/py | 3993/54949 | 4443/67120 | 4724/75951 | 4665/81742 | 4594/89376 |  |
| Model 1 | Ref. | 0.92 (0.88, 0.96) | 0.88 (0.85, 0.92) | 0.82 (0.78, 0.85) | 0.74 (0.71, 0.77) | <0.001 |
| Model 2 | Ref. | 0.97 (0.92, 1.00) | 0.97 (0.93, 1.02) | 0.93 (0.89, 0.98) | 0.88 (0.84, 0.93) | <0.001 |
| Flavanones |  |  |  |  |  |  |
| Intake (mg/d) | 4.8 [0.7 – 7.6] | 15.9 [12.3 – 21.6] | 35.8 [30.8 – 43.3] | 57.9 [55.0 – 61.5] | 83.6 [71.8 – 107.3] |  |
| Events/py | 4041/59927 | 4209/68610 | 4541/74361 | 4739/80803 | 4889/85438 |  |
| Model 1 | Ref. | 0.93 (0.89, 0.97) | 0.90 (0.87, 0.94) | 0.84 (0.80, 0.88) | 0.81 (0.77, 0.84) | <0.001 |
| Model 2 | Ref. | 0.96 (0.92, 1.00) | 0.95 (0.91, 0.99) | 0.90 (0.86, 0.94) | 0.89 (0.85, 0.93) | <0.001 |
| Flavones |  |  |  |  |  |  |
| Intake (mg/d) | 0.5 [0.4 – 0.7] | 1.0 [0.9 – 1.2] | 1.7 [1.5 – 1.9] | 2.4 [2.3 – 2.6] | 3.5 [3.1 – 4.2] |  |
| Events/py | 4033/56491 | 4281/68716 | 4514/73740 | 4865/83671 | 4726/86520 |  |
| Model 1 | Ref. | 0.89 (0.86, 0.93) | 0.86 (0.83, 0.90) | 0.82 (0.79, 0.86) | 0.77 (0.73, 0.80) | <0.001 |
| Model 2 | Ref. | 0.94 (0.90, 0.98) | 0.92 (0.88, 0.96) | 0.90 (0.86, 0.94) | 0.86 (0.82, 0.90) | <0.001 |
| Poor mental health | |  |  |  |  |  |
| Flavonols |  |  |  |  |  |  |
| Intake (mg/d) | 6.7 [5.4 – 7.8] | 10.7 [9.8 – 11.7] | 15.0 [13.8 – 16.2] | 21.4 [19.4 – 24.1] | 36.8 [32.0 – 46.0] |  |
| Events/py | 2016/117226 | 1925/129636 | 1701/136399 | 1731/136245 | 1571/133175 |  |
| Model 1 | Ref. | 0.90 (0.85, 0.96) | 0.79 (0.74, 0.84) | 0.83 (0.77, 0.88) | 0.79 (0.74, 0.84) | <0.001 |
| Model 2 | Ref. | 0.98 (0.91, 1.04) | 0.87 (0.81, 0.93) | 0.92 (0.86, 0.99) | 0.87 (0.80, 0.93) | 0.004 |
| Flavan-3-ols monomers | | |  |  |  |  |
| Intake (mg/d) | 7.1 [4.7 – 8.9] | 14.1 [12.4 – 15.9] | 23.7 [20.4 – 29.8] | 62.6 [44.2 – 73.2] | 174.4 [89.3 – 183.7] |  |
| Events/py | 1884/117847 | 1861/130743 | 1709/135566 | 1715/135522 | 1775/133003 |  |
| Model 1 | Ref. | 0.91 (0.85, 0.97) | 0.82 (0.77, 0.88) | 0.84 (0.78, 0.89) | 0.87 (0.82, 0.93) | 0.037 |
| Model 2 | Ref. | 1.00 (0.94, 1.07) | 0.95 (0.88, 1.01) | 0.95 (0.89, 1.02) | 0.98 (0.92, 1.05) | 0.919 |
| Flavan-3-ols polymers | | |  |  |  |  |
| Intake (mg/d) | 37.6 [26.4 – 47.7] | 80.4 [69.5 – 92.1] | 136.2 [119.3 – 155.3] | 231.0 [203.1 – 259.1] | 547.2 [377.6 – 626.8] |  |
| Events/py | 1874/117221 | 1821/130234 | 1811/135918 | 1745/135990 | 1693/133318 |  |
| Model 1 | Ref. | 0.90 (0.84, 0.95) | 0.87 (0.81, 0.92) | 0.84 (0.79, 0.90) | 0.84 (0.78, 0.89) | <0.001 |
| Model 2 | Ref. | 0.97 (0.90, 1.03) | 0.96 (0.89, 1.02) | 0.93 (0.86, 0.99) | 0.93 (0.86, 0.99) | 0.064 |
| Anthocyanins |  |  |  |  |  |  |
| Intake (mg/d) | 1.4 [0.6 – 2.1] | 3.8 [3.1 – 4.2] | 6.2 [5.4 – 8.0] | 14.1 [13.2 – 15.1] | 23.1 [21.2 – 28.5] |  |
| Events/py | 2055/112298 | 1854/129033 | 1804/134921 | 1693/137382 | 1538/139048 |  |
| Model 1 | Ref. | 0.82 (0.77, 0.87) | 0.79 (0.74, 0.84) | 0.74 (0.70, 0.79) | 0.68 (0.64, 0.73) | <0.001 |
| Model 2 | Ref. | 0.89 (0.84, 0.95) | 0.90 (0.84, 0.96) | 0.89 (0.83, 0.95) | 0.84 (0.78, 0.90) | <0.001 |
| Flavanones |  |  |  |  |  |  |
| Intake (mg/d) | 4.7 [0.6 – 7.6] | 15.9 [12.6 – 21.9] | 35.3 [30.8 – 43.0] | 57.9 [55.0 – 61.5] | 83.2 [71.7 – 107.2] |  |
| Events/py | 1965/116915 | 1629/121773 | 1794/130032 | 1796/141543 | 1760/142417 |  |
| Model 1 | Ref. | 0.83 (0.77, 0.88) | 0.83 (0.78, 0.88) | 0.73 (0.69, 0.78) | 0.72 (0.67, 0.77) | <0.001 |
| Model 2 | Ref. | 0.86 (0.81, 0.92) | 0.89 (0.83, 0.95) | 0.79 (0.74, 0.84) | 0.78 (0.72, 0.83) | <0.001 |
| Flavones |  |  |  |  |  |  |
| Intake (mg/d) | 0.5 [0.4 – 0.6] | 1.0 [0.9 – 1.2] | 1.7 [1.5 – 1.9] | 2.4 [2.3 – 2.6] | 3.5 [3.1 – 4.2] |  |
| Events/py | 1949/114510 | 1770/126017 | 1826/132007 | 1758/140707 | 1641/139440 |  |
| Model 1 | Ref. | 0.86 (0.80, 0.91) | 0.83 (0.78, 0.88) | 0.75 (0.70, 0.80) | 0.71 (0.66, 0.75) | <0.001 |
| Model 2 | Ref. | 0.92 (0.86, 0.98) | 0.89 (0.84, 0.96) | 0.83 (0.78, 0.89) | 0.77 (0.72, 0.83) | <0.001 |
| Hazard ratios (95% CI) for frailty, physical impairment and poor mental health during 24 years of follow up, obtained from Cox proportional hazards models. Model 1 adjusted for age and questionnaire cycle; Model 2 adjusted for age, questionnaire cycle, ethnicity, smoking status, marital status, menopausal status, family history of myocardial infarction, diabetes and cancer, multivitamin use, use of other medications, history of hypertension, hypercholesterolemia, diabetes, myocardial infarction, and stroke, physical activity, BMI, and intakes of alcohol, total energy, meat, nuts, saturated fat, polyunsaturated fat, trans fat, cereal fibre, and soft drink. Intakes are reported as median [p25 - p75]. Py, person years. | | | | | | |

| **Supplementary Table 3**. Associations between time-updated flavonoid subclass intakes and healthy aging domains in the Health Professionals Follow-up Study | | | | | | |
| --- | --- | --- | --- | --- | --- | --- |
|  | Quintiles of flavonoid intake | | | | | p-trend |
|  | Q1 | Q2 | Q3 | Q4 | Q5 |  |
| Frailty |  |  |  |  |  |  |
| Flavonols |  |  |  |  |  |  |
| Intake (mg/d) | 8.9 [7.0 – 10.3] | 13.9 [12.8 – 15.1] | 19.1 [17.8 – 20.7] | 26.3 [24.3 – 28.6] | 40.7 [35.6 – 49.5] |  |
| Events/py | 450/34048 | 438/39015 | 393 /41835 | 340 /41590 | 336 /41884 |  |
| Model 1 | Ref. | 0.92 (0.80, 1.05) | 0.85 (0.74, 0.97) | 0.75 (0.65, 0.86) | 0.76 (0.65, 0.88) | <0.001 |
| Model 2 | Ref. | 1.04 (0.90, 1.20) | 0.99 (0.85, 1.15) | 0.93 (0.79, 1.09) | 0.96 (0.81, 1.14) | 0.390 |
| Flavan-3-ols monomers | | |  |  |  |  |
| Intake (mg/d) | 10.3 [7.4 – 12.7] | 19.2 [17.0 – 21.5] | 29.1 [26.3 – 32.7] | 47.3 [41.9 – 54.3] | 90.6 [76.9 – 169.2] |  |
| Events/py | 464/34432 | 430/39143 | 390 /41051 | 337 /42273 | 336 /41474 |  |
| Model 1 | Ref. | 0.85 (0.74, 0.97) | 0.80 (0.69, 0.91) | 0.71 (0.62, 0.82) | 0.70 (0.60, 0.81) | <0.001 |
| Model 2 | Ref. | 1.04 (0.90, 1.19) | 1.04 (0.90, 1.21) | 0.96 (0.82, 1.12) | 0.96 (0.82, 1.12) | 0.296 |
| Flavan-3-ols polymers | | |  |  |  |  |
| Intake (mg/d) | 57.3 [41.4 – 70.2] | 107.8 [95.2 – 120.5] | 162.2 [147.5 – 176.6] | 234.3 [212.1 – 257.7] | 392.1 [330.1 – 567.8] |  |
| Events/py | 407/34423 | 428/39257 | 386 /40777 | 394 /41674 | 342 /42241 |  |
| Model 1 | Ref. | 0.95 (0.83, 1.10) | 0.83 (0.72, 0.96) | 0.84 (0.73, 0.97) | 0.72 (0.62, 0.83) | <0.001 |
| Model 2 | Ref. | 1.10 (0.95, 1.27) | 1.04 (0.89, 1.21) | 1.10 (0.94, 1.29) | 1.01 (0.85, 1.20) | 0.695 |
| Anthocyanins |  |  |  |  |  |  |
| Intake (mg/d) | 3.7 [2.2 – 5.2] | 11.0 [8.6 – 13.3] | 18.6 [16.7 – 20.6] | 28.2 [25.4 – 31.7] | 68.7 [50.5 – 91.1] |  |
| Events/py | 463/33616 | 413/37700 | 393 /40289 | 372 /42639 | 316 /44128 |  |
| Model 1 | Ref. | 0.82 (0.72, 0.94) | 0.79 (0.69, 0.91) | 0.73 (0.64, 0.84) | 0.61 (0.53, 0.70) | <0.001 |
| Model 2 | Ref. | 0.92 (0.80, 1.05) | 0.97 (0.84, 1.12) | 1.00 (0.86, 1.16) | 0.88 (0.75, 1.04) | 0.215 |
| Flavanones |  |  |  |  |  |  |
| Intake (mg/d) | 5.0 [1.4 – 7.9] | 16.6 [12.9 – 24.0] | 36.7 [31.8 – 45.4] | 59.4 [55.7 – 62.9] | 84.6 [73.6 – 111.8] |  |
| Events/py | 399/38204 | 353 /41347 | 392 /40462 | 423/38923 | 390 /39436 |  |
| Model 1 | Ref. | 0.83 (0.72, 0.96) | 0.85 (0.74, 0.98) | 0.82 (0.71, 0.95) | 0.76 (0.66, 0.88) | 0.001 |
| Model 2 | Ref. | 0.93 (0.80, 1.08) | 1.02 (0.88, 1.18) | 0.98 (0.85, 1.13) | 0.97 (0.83, 1.13) | 0.961 |
| Flavones |  |  |  |  |  |  |
| Intake (mg/d) | 0.9 [0.6 – 1.2] | 2.0 [1.7 – 2.2] | 2.8 [2.6 – 3.1] | 4.0 [3.6 – 4.4] | 6.9 [5.7 – 9.0] |  |
| Events/py | 467/39243 | 391 /41494 | 392 /41249 | 323 /39463 | 384/36922 |  |
| Model 1 | Ref. | 0.83 (0.72, 0.95) | 0.86 (0.75, 0.99) | 0.77 (0.67, 0.89) | 0.93 (0.81, 1.07) | 0.558 |
| Model 2 | Ref. | 0.93 (0.80, 1.07) | 1.02 (0.88, 1.18) | 0.95 (0.81, 1.11) | 1.06 (0.91, 1.23) | 0.300 |
| Impaired physical function | | |  |  |  |  |
| Flavonols |  |  |  |  |  |  |
| Intake (mg/d) | 9.0 [7.1 – 10.4] | 14.0 [12.8 – 15.2] | 19.2 [17.8 – 20.7] | 26.3 [24.3 – 28.6] | 40.8 [35.6 – 49.8] |  |
| Events/py | 774/21027 | 898/26695 | 847/29417 | 835/29198 | 811/30382 |  |
| Model 1 | Ref. | 0.98 (0.89, 1.08) | 0.87 (0.78, 0.96) | 0.87 (0.79, 0.96) | 0.83 (0.75, 0.92) | <0.001 |
| Model 2 | Ref. | 1.05 (0.94, 1.16) | 0.98 (0.88, 1.09) | 1.02 (0.91, 1.14) | 1.01 (0.90, 1.14) | 0.993 |
| Flavan-3-ols monomers | | |  |  |  |  |
| Intake (mg/d) | 10.4 [7.5 – 12.8] | 19.3 [17.1 – 21.5] | 29.2 [26.3 – 32.8] | 47.3 [41.7 – 54.3] | 91.7 [77.3 – 172.3] |  |
| Events/py | 821/21544 | 879/26070 | 826/28848 | 835/30545 | 804/29713 |  |
| Model 1 | Ref. | 0.88 (0.79, 0.97) | 0.81 (0.73, 0.89) | 0.80 (0.72, 0.88) | 0.77 (0.70, 0.85) | <0.001 |
| Model 2 | Ref. | 0.97 (0.88, 1.07) | 0.95 (0.85, 1.05) | 0.97 (0.87, 1.08) | 0.95 (0.85, 1.05) | 0.463 |
| Flavan-3-ols polymers | | |  |  |  |  |
| Intake (mg/d) | 57.8 [42.1 – 70.3] | 107.3 [95.1 – 119.9] | 162.6 [147.5 – 176.8] | 233.6 [212.1 – 257.4] | 391.9 [330.3 – 571.7] |  |
| Events/py | 790/21848 | 867/26556 | 870/28459 | 811/29457 | 827/30400 |  |
| Model 1 | Ref. | 0.88 (0.80, 0.98) | 0.84 (0.76, 0.93) | 0.75 (0.67, 0.82) | 0.75 (0.68, 0.83) | <0.001 |
| Model 2 | Ref. | 0.95 (0.86, 1.05) | 0.94 (0.85, 1.05) | 0.88 (0.78, 0.98) | 0.95 (0.84, 1.06) | 0.693 |
| Anthocyanins |  |  |  |  |  |  |
| Intake (mg/d) | 3.7 [2.3 – 5.2] | 11.1 [8.6 – 13.3] | 18.6 [16.8 – 20.6] | 28.3 [25.4 – 31.7] | 68.9 [50.2 – 90.4] |  |
| Events/py | 809/20791 | 846/24719 | 824/28052 | 841/30714 | 845/32444 |  |
| Model 1 | Ref. | 0.89 (0.80, 0.98) | 0.82 (0.74, 0.90) | 0.77 (0.69, 0.85) | 0.71 (0.64, 0.79) | <0.001 |
| Model 2 | Ref. | 0.93 (0.84, 1.03) | 0.93 (0.84, 1.04) | 0.93 (0.84, 1.04) | 0.92 (0.82, 1.02) | 0.284 |
| Flavanones |  |  |  |  |  |  |
| Intake (mg/d) | 5.1 [1.7 – 7.9] | 16.5 [12.9 – 23.7] | 36.4 [31.7 – 45.2] | 59.6 [55.7 – 63.0] | 84.8 [73.5 – 112.4] |  |
| Events/py | 790/25527 | 862/29388 | 884/27904 | 826/26491 | 803/27411 |  |
| Model 1 | Ref. | 0.96 (0.87, 1.06) | 0.95 (0.86, 1.05) | 0.87 (0.78, 0.96) | 0.80 (0.73, 0.89) | <0.001 |
| Model 2 | Ref. | 1.00 (0.9, 1.11) | 1.05 (0.94, 1.16) | 0.97 (0.87, 1.07) | 0.92 (0.83, 1.03) | 0.068 |
| Flavones |  |  |  |  |  |  |
| Intake (mg/d) | 0.9 [0.6 – 1.2] | 1.9 [1.7 – 2.2] | 2.9 [2.6 – 3.1] | 4.0 [3.6 – 4.4] | 6.8 [5.7 – 9.0] |  |
| Events/py | 936/25732 | 872/28286 | 870/28958 | 732/27895 | 755/25849 |  |
| Model 1 | Ref. | 0.87 (0.79, 0.95) | 0.90 (0.82, 0.99) | 0.80 (0.72, 0.88) | 0.89 (0.80, 0.98) | 0.029 |
| Model 2 | Ref. | 0.94 (0.85, 1.03) | 0.98 (0.89, 1.08) | 0.92 (0.83, 1.02) | 0.96 (0.86, 1.07) | 0.580 |
| Poor mental health | |  |  |  |  |  |
| Flavonols |  |  |  |  |  |  |
| Intake (mg/d) | 8.9 [7.0 – 10.3] | 13.9 [12.8 – 15.1] | 19.1 [17.7 – 20.7] | 26.3 [24.2 – 28.6] | 40.6 [35.6 – 49.6] |  |
| Events/py | 376/34142 | 368/39264 | 313/41848 | 309/41793 | 303/41817 |  |
| Model 1 | Ref. | 0.96 (0.83, 1.11) | 0.77 (0.66, 0.90) | 0.79 (0.68, 0.92) | 0.80 (0.68, 0.93) | 0.002 |
| Model 2 | Ref. | 1.03 (0.89, 1.21) | 0.84 (0.71, 0.98) | 0.89 (0.75, 1.05) | 0.87 (0.72, 1.05) | 0.093 |
| Flavan-3-ols monomers | | |  |  |  |  |
| Intake (mg/d) | 10.3 [7.3 – 12.7] | 19.2 [17.0 – 21.5] | 29.1 [26.3 – 32.7] | 47.2 [41.8 – 54.3] | 90.4 [76.9 – 168.1] |  |
| Events/py | 371/34762 | 355/39445 | 313/41006 | 312/42371 | 318/41279 |  |
| Model 1 | Ref. | 0.86 (0.74, 1.00) | 0.80 (0.69, 0.94) | 0.79 (0.67, 0.92) | 0.80 (0.68, 0.93) | 0.020 |
| Model 2 | Ref. | 0.97 (0.83, 1.13) | 0.93 (0.79, 1.10) | 0.92 (0.78, 1.09) | 0.92 (0.78, 1.09) | 0.408 |
| Flavan-3-ols polymers | | |  |  |  |  |
| Intake (mg/d) | 57.2 [41.2 – 70.1] | 107.6 [95.1 – 120.4] | 162.0 [147.2 – 176.5] | 234.3 [212.1 – 257.7] | 390.1 [328.4 – 564.8] |  |
| Events/py | 351/34590 | 312/39417 | 366/40854 | 298/41854 | 342/42149 |  |
| Model 1 | Ref. | 0.81 (0.69, 0.94) | 0.92 (0.79, 1.07) | 0.73 (0.62, 0.85) | 0.83 (0.72, 0.97) | 0.108 |
| Model 2 | Ref. | 0.86 (0.73, 1.01) | 1.04 (0.89, 1.22) | 0.81 (0.68, 0.97) | 0.95 (0.80, 1.14) | 0.939 |
| Anthocyanins |  |  |  |  |  |  |
| Intake (mg/d) | 3.7 [2.2 – 5.2] | 11.1 [8.6 – 13.3] | 18.5 [16.7 – 20.5] | 28.2 [25.3 – 31.7] | 68.7 [50.4 – 90.3] |  |
| Events/py | 367/33872 | 376/37844 | 329/40395 | 301/42722 | 296/44031 |  |
| Model 1 | Ref. | 0.93 (0.80, 1.08) | 0.84 (0.72, 0.98) | 0.72 (0.62, 0.85) | 0.69 (0.59, 0.81) | <0.001 |
| Model 2 | Ref. | 0.98 (0.84, 1.14) | 0.94 (0.80, 1.11) | 0.86 (0.72, 1.01) | 0.82 (0.69, 0.97) | 0.016 |
| Flavanones |  |  |  |  |  |  |
| Intake (mg/d) | 5.0 [1.4 – 7.9] | 16.6 [12.9 – 23.9] | 36.8 [31.8 – 45.5] | 59.4 [55.7 – 62.9] | 84.6 [73.5 – 112.2] |  |
| Events/py | 349/38303 | 342/41048 | 319/40516 | 336/39365 | 323/39632 |  |
| Model 1 | Ref. | 0.94 (0.80, 1.09) | 0.85 (0.73, 0.99) | 0.84 (0.72, 0.98) | 0.78 (0.67, 0.92) | 0.001 |
| Model 2 | Ref. | 1.02 (0.87, 1.19) | 0.95 (0.81, 1.12) | 0.94 (0.80, 1.10) | 0.90 (0.76, 1.06) | 0.111 |
| Flavones |  |  |  |  |  |  |
| Intake (mg/d) | 0.9 [0.6 – 1.2] | 1.9 [1.7 – 2.2] | 2.8 [2.6 – 3.1] | 4.0 [3.6 – 4.4] | 6.9 [5.8 – 9.0] |  |
| Events/py | 412/39157 | 373/41473 | 322/41327 | 257/39566 | 305/37341 |  |
| Model 1 | Ref. | 0.87 (0.75, 1.00) | 0.80 (0.69, 0.93) | 0.69 (0.58, 0.80) | 0.83 (0.72, 0.97) | 0.011 |
| Model 2 | Ref. | 0.93 (0.80, 1.07) | 0.86 (0.74, 1.01) | 0.75 (0.63, 0.90) | 0.86 (0.73, 1.02) | 0.073 |
| Hazard ratios (95% CI) for frailty, physical impairment and poor mental health during 12 years of follow up, obtained from Cox proportional hazards models. Model 1 adjusted for age and questionnaire cycle; Model 2 adjusted for age, questionnaire cycle, ethnicity, smoking status, marital status, family history of myocardial infarction, diabetes and cancer, multivitamin use, use of aspirin, use of other medications, history of hypertension, hypercholesterolemia, diabetes, myocardial infarction, and stroke, physical activity, BMI, and intakes of alcohol, total energy, meat, nuts, saturated fat, polyunsaturated fat, trans fat, cereal fibre, and soft drink. Intakes are reported as median [IQR]. Py, person years. | | | | | | |

| **Supplementary Table 4**. Associations between 4-year changes in intake of flavonoid-rich foods and healthy aging domains in the Health Professionals Follow-up Study | | | | | | | | | |
| --- | --- | --- | --- | --- | --- | --- | --- | --- | --- |
|  | 4-year change in intake levels of the flavodiet score, servings/wk | | | | | | |  |  |
|  |  | Decrease |  |  |  | Increase |  |  |  |
|  | ≥7 | 4-6.9 | 1-3.9 | No change (±<1) | 1-3.9 | 4-6.9 | ≥7 | P_trend_ | Every  3 servings/d change |
| Frailty |  |  |  |  |  |  |  |  |  |
| Events/py | 298/25404 | 207/18249 | 335/35196 | 454/43474 | 309/33965 | 149/18226 | 205/23858 |  |  |
| Model 1 | 1.31 (1.10, 1.56) | 1.13 (0.95, 1.35) | 0.90 (0.77, 1.03) | Ref. | 0.95 (0.82, 1.10) | 0.90 (0.74, 1.08) | 0.96 (0.81, 1.14) | 0.030 | 0.85 (0.73, 0.98) |
| Model 2 | 1.09 (0.91, 1.31) | 1.09 (0.92, 1.30) | 0.88 (0.76, 1.02) | Ref. | 0.98 (0.84, 1.14) | 0.90 (0.74, 1.10) | 0.99 (0.83, 1.18) | 0.950 | 1.00 (0.86, 1.15) |
| Impaired physical function | |  |  |  |  |  |  |  |  |
| Events/py | 564/17090 | 409/12414 | 746/24165 | 958/29449 | 677/24093 | 377/12747 | 434/16761 |  |  |
| Model 1 | 1.11 (0.98, 1.25) | 1.01 (0.90, 1.14) | 0.94 (0.85, 1.04) | Ref. | 0.92 (0.83, 1.02) | 0.99 (0.88, 1.12) | 0.96 (0.85, 1.08) | 0.077 | 0.91 (0.82, 1.01) |
| Model 2 | 0.99 (0.87, 1.12) | 0.96 (0.85, 1.09) | 0.91 (0.82, 1.01) | Ref. | 0.92 (0.83, 1.02) | 0.99 (0.88, 1.13) | 0.99 (0.88, 1.12) | 0.620 | 1.03 (0.93, 1.14) |
| Poor mental health | |  |  |  |  |  |  |  |  |
| Events/py | 267/25264 | 167/18514 | 296/35332 | 362/43583 | 255/34013 | 150/18251 | 172/23907 |  |  |
| Model 1 | 1.70 (1.41, 2.05) | 1.17 (0.97, 1.42) | 1.06 (0.90, 1.24) | Ref. | 1.04 (0.89, 1.23) | 1.13 (0.93, 1.37) | 1.07 (0.89, 1.29) | 0.003 | 0.78 (0.66, 0.92) |
| Model 2 | 1.60 (1.32, 1.95) | 1.19 (0.98, 1.45) | 1.10 (0.93, 1.29) | Ref. | 1.11 (0.94, 1.31) | 1.17 (0.96, 1.43) | 1.12 (0.92, 1.35) | 0.044 | 0.85 (0.72, 1.00) |
| 4-year change in intake levels of flavonoid-rich foods and beverages, servings/wk | | | | | | | | | |
|  |  | Decrease |  |  |  | Increase |  |  |  |
|  | ≥2 | 1-1.99^#^ | 0.5-0.99 | No change (±0.49) | 0.5-0.99 | 1-1.99^#^ | ≥2 | P_trend_ | Every  3.5 servings/wk change^*^ |
| Frailty |  |  |  |  |  |  |  |  |  |
| Tea |  |  |  |  |  |  |  |  |  |
| Events/py | 341/35341 | 34/3946 | 85/7626 | 1136/110058 | 46/6248 | 42/3823 | 273/31329 |  |  |
| Model 1 | 0.93 (0.81, 1.08) | 0.90 (0.64, 1.28) | 1.21 (0.97, 1.52) | Ref. | 0.86 (0.64, 1.17) | 1.15 (0.84, 1.58) | 1.01 (0.88, 1.15) | 0.692 | 1.01 (0.95, 1.09) |
| Model 2 | 0.90 (0.78, 1.04) | 0.91 (0.64, 1.30) | 1.18 (0.94, 1.48) | Ref. | 0.84 (0.62, 1.15) | 1.15 (0.83, 1.58) | 0.99 (0.86, 1.13) | 0.719 | 1.01 (0.95, 1.09) |
| Red wine | |  |  |  |  |  |  |  |  |
| Events/py |  | 216/22971 | 30/2402 | 1533/147265 | 24/2084 | 154/23648 |  |  |  |
| Model 1 |  | 1.01 (0.86, 1.20) | 1.19 (0.82, 1.72) | Ref. | 1.39 (0.92, 2.10) | 0.78 (0.65, 0.92) |  | 0.006 | 0.89 (0.82, 0.97) |
| Model 2 |  | 0.94 (0.78, 1.14) | 1.13 (0.77, 1.65) | Ref. | 1.26 (0.82, 1.93) | 0.91 (0.76, 1.10) |  | 0.748 | 0.99 (0.90, 1.07) |
| Blueberry | | |  |  |  |  |  |  |  |
| Events/py |  | 132/11704 | 17/1732 | 1508/152154 | 41/4802 | 259/27980 |  |  |  |
| Model 1 |  | 1.02 (0.82, 1.27) | 0.90 (0.55, 1.46) | Ref. | 0.96 (0.7, 1.32) | 0.81 (0.70, 0.93) |  | 0.022 | 0.89 (0.80, 0.98) |
| Model 2 |  | 1.02 (0.82, 1.28) | 0.85 (0.52, 1.39) | Ref. | 1.00 (0.73, 1.38) | 0.90 (0.78, 1.04) |  | 0.900 | 0.99 (0.90, 1.10) |
| Apple |  |  |  |  |  |  |  |  |  |
| Events/py | 403/37642 | 18/2532 | 29/1922 | 1194/120379 | 19/1847 | 21/2164 | 273/31886 |  |  |
| Model 1 | 1.22 (1.07, 1.40) | 1.04 (0.64, 1.70) | 1.34 (0.92, 1.95) | Ref. | 1.07 (0.67, 1.69) | 1.28 (0.81, 2.02) | 0.92 (0.80, 1.05) | 0.006 | 0.89 (0.81, 0.97) |
| Model 2 | 1.18 (1.03, 1.35) | 0.96 (0.58, 1.58) | 1.25 (0.85, 1.84) | Ref. | 0.95 (0.59, 1.52) | 1.30 (0.82, 2.06) | 0.98 (0.85, 1.12) | 0.246 | 0.95 (0.87, 1.04) |
| Strawberry | | | |  |  |  |  |  |  |
| Events/py |  | 136/12373 | 40/2410 | 1511/156862 | 44/4116 | 226/22611 |  |  |  |
| Model 1 |  | 1.14 (0.91, 1.43) | 1.50 (1.08, 2.08) | Ref. | 1.13 (0.83, 1.54) | 0.93 (0.80, 1.07) |  | 0.087 | 0.89 (0.79, 1.02) |
| Model 2 |  | 1.09 (0.86, 1.37) | 1.51 (1.08, 2.10) | Ref. | 1.06 (0.77, 1.45) | 0.98 (0.85, 1.14) |  | 0.514 | 0.96 (0.84, 1.09) |
| Orange | | | |  |  |  |  |  |  |
| Events/py | 323/30612 | 17/1210 | 32/3906 | 1341/135350 | 24/2478 | 6/974 | 214/23842 |  |  |
| Model 1 | 1.06 (0.91, 1.22) | 1.10 (0.66, 1.84) | 0.75 (0.52, 1.07) | Ref. | 0.99 (0.66, 1.50) | 0.58 (0.25, 1.32) | 0.97 (0.84, 1.13) | 0.921 | 1.01 (0.92, 1.10) |
| Model 2 | 1.03 (0.89, 1.20) | 1.07 (0.64, 1.80) | 0.75 (0.52, 1.07) | Ref. | 0.98 (0.64, 1.48) | 0.51 (0.22, 1.19) | 1.00 (0.86, 1.16) | 0.462 | 1.03 (0.95, 1.13) |
| Grapefruit and grapefruit juice | | |  |  |  |  |  |  |  |
| Events/py | 185/14430 | 92/8887 | 102/8560 | 1439/154382 | 24/2651 | 50/4925 | 65/4536 |  |  |
| Model 1 | 1.12 (0.95, 1.31) | 0.90 (0.72, 1.12) | 1.18 (0.96, 1.44) | Ref. | 0.95 (0.63, 1.44) | 0.92 (0.68, 1.24) | 1.37 (1.03, 1.81) | 0.720 | 1.02 (0.90, 1.17) |
| Model 2 | 1.18 (1.01, 1.39) | 1.02 (0.82, 1.28) | 1.22 (0.99, 1.51) | Ref. | 0.96 (0.63, 1.45) | 0.95 (0.70, 1.29) | 1.30 (0.98, 1.73) | 0.624 | 0.97 (0.85, 1.10) |
| Impaired physical function | |  |  |  |  |  |  |  |  |
| Tea |  |  |  |  |  |  |  |  |  |
| Events/py | 748/24289 | 83/2761 | 149/5320 | 2413/75408 | 108/4293 | 81/2690 | 583/21959 |  |  |
| Model 1 | 0.99 (0.90, 1.09) | 1.02 (0.82, 1.28) | 0.93 (0.79, 1.10) | Ref. | 1.00 (0.82, 1.22) | 1.04 (0.82, 1.30) | 0.99 (0.90, 1.09) | 0.761 | 0.99 (0.95, 1.04) |
| Model 2 | 0.98 (0.89, 1.08) | 1.04 (0.83, 1.30) | 0.92 (0.78, 1.09) | Ref. | 0.93 (0.76, 1.14) | 1.05 (0.83, 1.32) | 0.99 (0.90, 1.09) | 0.983 | 1.00 (0.95, 1.05) |
| Red wine |  |  |  |  |  |  |  |  |  |
| Events/py |  | 514/16098 | 60/1504 | 3125/100037 | 40/1407 | 426/17674 |  |  |  |
| Model 1 |  | 1.06 (0.95, 1.18) | 1.22 (0.94, 1.59) | Ref. | 1.06 (0.76, 1.46) | 0.91 (0.82, 1.00) |  | 0.034 | 0.95 (0.90, 1.00) |
| Model 2 |  | 1.04 (0.92, 1.17) | 1.14 (0.87, 1.50) | Ref. | 0.99 (0.71, 1.38) | 1.00 (0.88, 1.12) |  | 0.679 | 0.99 (0.94, 1.04) |
| Blueberry | | |  |  |  |  |  |  |  |
| Events/py |  | 292/8249 | 47/1196 | 3060/103778 | 79/3341 | 687/20156 |  |  |  |
| Model 1 |  | 1.06 (0.91, 1.23) | 1.11 (0.82, 1.49) | Ref. | 0.84 (0.67, 1.06) | 0.98 (0.9, 1.06) |  | 0.134 | 0.95 (0.89, 1.02) |
| Model 2 |  | 1.03 (0.88, 1.19) | 1.06 (0.78, 1.42) | Ref. | 0.86 (0.68, 1.09) | 1.05 (0.96, 1.14) |  | 0.456 | 1.03 (0.96, 1.10) |
| Apple |  |  |  |  |  |  |  |  |  |
| Events/py | 815/25872 | 40/1870 | 41/1277 | 2524/82528 | 40/1272 | 40/1596 | 665/22304 |  |  |
| Model 1 | 1.09 (0.99, 1.19) | 0.88 (0.63, 1.22) | 1.00 (0.73, 1.39) | Ref. | 1.06 (0.77, 1.45) | 1.02 (0.74, 1.41) | 0.97 (0.89, 1.06) | 0.217 | 0.97 (0.91, 1.02) |
| Model 2 | 1.03 (0.94, 1.13) | 0.81 (0.58, 1.13) | 1.01 (0.73, 1.40) | Ref. | 1.02 (0.74, 1.40) | 1.07 (0.77, 1.48) | 1.00 (0.91, 1.09) | 0.638 | 1.01 (0.96, 1.07) |
| Strawberry | | |  |  |  |  |  |  |  |
| Events/py |  | 325/8644 | 70/1608 | 3194/107964 | 95/2832 | 481/15671 |  |  |  |
| Model 1 |  | 1.18 (1.02, 1.37) | 1.23 (0.96, 1.57) | Ref. | 1.23 (0.99, 1.52) | 0.91 (0.82, 1.01) |  | 0.008 | 0.89 (0.81, 0.97) |
| Model 2 |  | 1.12 (0.96, 1.30) | 1.24 (0.97, 1.58) | Ref. | 1.23 (0.99, 1.52) | 0.95 (0.86, 1.05) |  | 0.225 | 0.95 (0.87, 1.03) |
| Orange | | |  |  |  |  |  |  |  |
| Events/py | 673/20648 | 24/824 | 65/2719 | 2874/93939 | 50/1714 | 18/679 | 461/16197 |  |  |
| Model 1 | 1.05 (0.94, 1.16) | 0.79 (0.51, 1.21) | 0.70 (0.54, 0.90) | Ref. | 1.06 (0.80, 1.41) | 0.86 (0.53, 1.39) | 1.04 (0.94, 1.15) | 0.719 | 1.01 (0.95, 1.08) |
| Model 2 | 1.01 (0.91, 1.13) | 0.74 (0.48, 1.13) | 0.71 (0.55, 0.91) | Ref. | 1.09 (0.82, 1.46) | 0.76 (0.47, 1.23) | 1.06 (0.95, 1.17) | 0.184 | 1.05 (0.98, 1.12) |
| Grapefruit and grapefruit juice | | | |  |  |  |  |  |  |
| Events/py | 305/9441 | 200/6243 | 191/5815 | 3200/106946 | 46/1853 | 121/3393 | 102/3028 |  |  |
| Model 1 | 0.95 (0.84, 1.08) | 0.97 (0.83, 1.12) | 1.03 (0.88, 1.2) | Ref. | 0.81 (0.60, 1.09) | 1.25 (1.03, 1.52) | 1.33 (1.06, 1.66) | 0.022 | 1.12 (1.02, 1.24) |
| Model 2 | 1.00 (0.88, 1.13) | 1.00 (0.86, 1.17) | 1.09 (0.94, 1.27) | Ref. | 0.86 (0.64, 1.16) | 1.28 (1.05, 1.55) | 1.34 (1.07, 1.68) | 0.087 | 1.09 (0.99, 1.21) |
| Poor mental health | | |  |  |  |  |  |  |  |
| Tea |  |  |  |  |  |  |  |  |  |
| Events/py | 317/35343 | 26/4029 | 72/7640 | 956/110302 | 43/6224 | 23/3893 | 232/31434 |  |  |
| Model 1 | 1.08 (0.93, 1.25) | 0.81 (0.54, 1.20) | 1.12 (0.87, 1.42) | Ref. | 0.99 (0.72, 1.34) | 0.77 (0.51, 1.18) | 0.98 (0.85, 1.14) | 0.029 | 0.92 (0.85, 0.99) |
| Model 2 | 1.06 (0.91, 1.24) | 0.83 (0.55, 1.23) | 1.11 (0.87, 1.42) | Ref. | 0.92 (0.67, 1.26) | 0.79 (0.52, 1.21) | 0.97 (0.84, 1.13) | 0.027 | 0.92 (0.85, 0.99) |
| Red wine |  |  |  |  |  |  |  |  |  |
| Events/py |  | 190/22936 | 26/2437 | 1272/147886 | 11/2106 | 170/23499 |  |  |  |
| Model 1 |  | 1.14 (0.96, 1.36) | 1.25 (0.84, 1.86) | Ref. | 0.71 (0.39, 1.29) | 1.01 (0.86, 1.19) |  | 0.643 | 0.98 (0.90, 1.06) |
| Model 2 |  | 1.11 (0.91, 1.35) | 1.24 (0.83, 1.85) | Ref. | 0.69 (0.37, 1.26) | 1.15 (0.96, 1.39) |  | 0.320 | 1.04 (0.96, 1.14) |
| Blueberry | | | |  |  |  |  |  |  |
| Events/py |  | 118/11607 | 9/1750 | 1284/152673 | 33/4797 | 225/28036 |  |  |  |
| Model 1 |  | 1.11 (0.87, 1.40) | 0.54 (0.28, 1.04) | Ref. | 0.87 (0.61, 1.23) | 0.84 (0.73, 0.97) |  | 0.004 | 0.85 (0.76, 0.95) |
| Model 2 |  | 1.09 (0.86, 1.38) | 0.55 (0.28, 1.06) | Ref. | 0.85 (0.59, 1.20) | 0.90 (0.77, 1.04) |  | 0.080 | 0.91 (0.81, 1.01) |
| Apple |  |  |  |  |  |  |  |  |  |
| Events/py | 346/37778 | 21/2520 | 19/1983 | 1004/120563 | 20/1850 | 9/2189 | 250/31980 |  |  |
| Model 1 | 1.21 (1.04, 1.40) | 1.37 (0.87, 2.17) | 1.00 (0.63, 1.59) | Ref. | 1.22 (0.77, 1.91) | 0.70 (0.36, 1.37) | 1.00 (0.87, 1.15) | 0.028 | 0.90 (0.83, 0.99) |
| Model 2 | 1.20 (1.03, 1.39) | 1.34 (0.84, 2.12) | 0.96 (0.60, 1.53) | Ref. | 1.16 (0.73, 1.84) | 0.73 (0.37, 1.42) | 1.04 (0.90, 1.20) | 0.174 | 0.94 (0.86, 1.03) |
| Strawberry | | |  |  |  |  |  |  |  |
| Events/py |  | 105/12431 | 19/2452 | 1324/156958 | 30/4174 | 191/22849 |  |  |  |
| Model 1 |  | 0.99 (0.77, 1.28) | 0.73 (0.45, 1.17) | Ref. | 0.97 (0.67, 1.40) | 0.93 (0.79, 1.08) |  | 0.443 | 0.95 (0.83, 1.09) |
| Model 2 |  | 0.97 (0.75, 1.25) | 0.70 (0.43, 1.13) | Ref. | 0.98 (0.68, 1.42) | 0.98 (0.83, 1.15) |  | 0.978 | 1.00 (0.87, 1.15) |
| Orange | | |  |  |  |  |  |  |  |
| Events/py | 255/30705 | 16/1232 | 38/3911 | 1136/135666 | 21/2466 | 4/981 | 199/23903 |  |  |
| Model 1 | 1.06 (0.90, 1.25) | 1.47 (0.86, 2.51) | 1.15 (0.82, 1.59) | Ref. | 1.08 (0.69, 1.67) | 0.53 (0.20, 1.44) | 1.10 (0.95, 1.29) | 0.754 | 1.03 (0.94, 1.14) |
| Model 2 | 1.06 (0.90, 1.26) | 1.54 (0.90, 2.64) | 1.12 (0.81, 1.57) | Ref. | 1.04 (0.67, 1.61) | 0.56 (0.21, 1.52) | 1.13 (0.97, 1.33) | 0.817 | 1.05 (0.95, 1.15) |
| Grapefruit and grapefruit juice | | |  |  |  |  |  |  |  |
| Events/py | 147/14696 | 75/9042 | 78/8651 | 1258/154249 | 23/2721 | 44/4903 | 44/4600 |  |  |
| Model 1 | 1.07 (0.90, 1.28) | 0.90 (0.71, 1.14) | 1.05 (0.83, 1.32) | Ref. | 1.03 (0.68, 1.58) | 1.06 (0.77, 1.46) | 1.14 (0.81, 1.59) | 0.524 | 1.02 (0.88, 1.19) |
| Model 2 | 1.11 (0.93, 1.33) | 0.98 (0.77, 1.25) | 1.08 (0.85, 1.37) | Ref. | 1.00 (0.65, 1.53) | 1.14 (0.83, 1.57) | 1.12 (0.80, 1.57) | 0.351 | 0.98 (0.85, 1.14) |
| Hazard ratios (95% CI) for frailty, physical impairment and poor mental health during 12 years of follow up, obtained from Cox proportional hazards models. Model 1 adjusted for baseline age, questionnaire cycle and intakes of the exposure variable of interest; Model 2 adjusted for baseline age, questionnaire cycle, ethnicity, smoking status, change in smoking status, marital status, family history of myocardial infarction, diabetes and cancer, multivitamin use, use of aspirin, use of other medications, history of hypertension, hypercholesterolemia, diabetes, myocardial infarction, and stroke, physical activity, change in physical activity, BMI, change in BMI, intakes of the exposure variable of interest, and both intakes and change in intakes of alcohol, total energy, meat, nuts, saturated fat, polyunsaturated fat, trans fat, cereal fibre, and soft drink.  *Except for tea, where the hazard ratio (95%CI) is presented for a 4-year change in intake of 1 serve per day. ^#^Except for red wine and strawberries where this is ≥1.  Py, person years. | | | | | | | | | |

| **Supplementary Table 5**. Associations between time-updated flavodiet and total flavonoid intakes and healthy aging domains with death included as an event | | | | | | |
| --- | --- | --- | --- | --- | --- | --- |
|  | Quintiles of intake | | | | | p-trend |
|  | Q1 | Q2 | Q3 | Q4 | Q5 |  |
| NHS |  |  |  |  |  |  |
| Frailty | |  |  |  |  |  |
| Flavodiet score | Ref. | 0.97 (0.91, 1.00) | 0.96 (0.89, 0.98) | 0.88 (0.83, 0.93) | 0.87 (0.82, 0.91) | <0.001 |
| Total flavonoids | Ref. | 0.96 (0.91, 1.00) | 0.95 (0.90, 1.00) | 0.91 (0.86, 0.96) | 0.88 (0.83, 0.94) | <0.001 |
| Impaired physical function | |  |  |  |  |  |
| Flavodiet score | Ref. | 0.93 (0.90, 0.97) | 0.91 (0.88, 0.95) | 0.88 (0.84, 0.92) | 0.88 (0.83, 0.90) | <0.001 |
| Total flavonoids | Ref. | 0.98 (0.94, 1.02) | 0.95 (0.91, 0.99) | 0.90 (0.86, 0.94) | 0.89 (0.85, 0.92) | <0.001 |
| Poor mental health | |  |  |  |  |  |
| Flavodiet score | Ref. | 0.96 (0.90, 1.01) | 0.94 (0.88, 1.00) | 0.88 (0.83, 0.93) | 0.89 (0.83, 0.94) | <0.001 |
| Total flavonoids | Ref. | 0.93 (0.87, 0.98) | 0.94 (0.89, 1.00) | 0.92 (0.86, 0.98) | 0.90 (0.85, 0.96) | 0.020 |
| HPFS |  |  |  |  |  |  |
| Frailty |  |  |  |  |  |  |
| Flavodiet score | Ref. | 0.98 (0.89, 1.08) | 0.92 (0.83, 1.01) | 0.98 (0.89, 1.09) | 1.00 (0.89, 1.11) | 0.498 |
| Total flavonoids | Ref. | 0.92 (0.84, 1.02) | 0.93 (0.84, 1.04) | 1.00 (0.90, 1.11) | 0.97 (0.87, 1.10) | 0.709 |
| Impaired physical function | |  |  |  |  |  |
| Flavodiet score | Ref. | 0.95 (0.87, 1.04) | 0.90 (0.82, 0.98) | 0.95 (0.86, 1.04) | 0.95 (0.86, 1.04) | 0.836 |
| Total flavonoids | Ref. | 0.94 (0.86, 1.03) | 0.93 (0.84, 1.02) | 0.89 (0.80, 0.98) | 0.93 (0.84, 1.04) | 0.333 |
| Poor mental health | |  |  |  |  |  |
| Flavodiet score | Ref. | 0.89 (0.80, 0.97) | 0.91 (0.83, 1.00) | 0.95 (0.86, 1.05) | 0.92 (0.83, 1.03) | 0.340 |
| Total flavonoids | Ref. | 0.89 (0.81, 0.99) | 0.93 (0.84, 1.04) | 0.96 (0.86, 1.07) | 1.01 (0.90, 1.13) | 0.237 |
| Hazard ratios (95% CI) for frailty, physical impairment and poor mental health obtained from Cox proportional hazards models adjusted for baseline age, questionnaire cycle, ethnicity, smoking status, marital status, menopausal status (for NHS only), family history of myocardial infarction, diabetes and cancer, multivitamin use, use of aspirin, use of other medications, history of hypertension, hypercholesterolemia, diabetes, myocardial infarction, and stroke, physical activity, BMI, and intakes of alcohol, total energy, meat, nuts, saturated fat, polyunsaturated fat, trans fat, cereal fibre, and soft drink (Model 2). | | | | | | |

| **Supplementary Table 6.** Associations between time-updated flavodiet and total flavonoid intakes and healthy aging domains in the first 12 years of follow-up in the Nurses’ Health Study | | | | | | |
| --- | --- | --- | --- | --- | --- | --- |
|  | Quintiles of intake | | | | | p-trend |
|  | Q1 | Q2 | Q3 | Q4 | Q5 |  |
| Frailty (n events = 3628) | |  |  |  |  |  |
| Flavodiet score | Ref. | 0.91 (0.83, 1.01) | 0.97 (0.88, 1.07) | 0.83 (0.74, 0.92) | 0.93 (0.84, 1.03) | 0.691 |
| Total flavonoids | Ref. | 0.92 (0.83, 1.02) | 0.87 (0.78, 0.97) | 0.86 (0.77, 0.96) | 0.84 (0.75, 0.94) | 0.016 |
| Impaired physical function (n events = 14867) | | |  |  |  |  |
| Flavodiet score | Ref. | 0.95 (0.90, 0.99) | 0.93 (0.88, 0.98) | 0.91 (0.86, 0.96) | 0.91 (0.86, 0.96) | 0.005 |
| Total flavonoids | Ref. | 0.95 (0.90, 1.00) | 0.95 (0.90, 1.00) | 0.93 (0.88, 0.98) | 0.91 (0.86, 0.96) | 0.006 |
| Poor mental health (n events = 6971) | |  |  |  |  |  |
| Flavodiet score | Ref. | 0.98 (0.91, 1.05) | 0.99 (0.92, 1.07) | 0.91 (0.84, 0.98) | 0.94 (0.87, 1.02) | 0.109 |
| Total flavonoids | Ref. | 0.93 (0.86, 1.01) | 0.98 (0.90, 1.05) | 0.93 (0.86, 1.01) | 0.94 (0.87, 1.02) | 0.341 |
| Hazard ratios (95% CI) for frailty, physical impairment and poor mental health obtained from Cox proportional hazards models adjusted for baseline age, questionnaire cycle, ethnicity, smoking status, marital status, menopausal status, family history of myocardial infarction, diabetes and cancer, multivitamin use, use of aspirin, use of other medications, history of hypertension, hypercholesterolemia, diabetes, myocardial infarction, and stroke, physical activity, BMI, and intakes of alcohol, total energy, meat, nuts, saturated fat, polyunsaturated fat, trans fat, cereal fibre, and soft drink (Model 2). | | | | | | |

| **Supplementary Table 7.** Associations between time-updated flavodiet and total flavonoid intakes and healthy aging domains in the Nurses’ Health Study with participants only entering the study at 70 years of age. | | | | | | |
| --- | --- | --- | --- | --- | --- | --- |
|  | Quintiles of intake | | | | | p-trend |
|  | Q1 | Q2 | Q3 | Q4 | Q5 |  |
| Frailty (n events = 8779) | |  |  |  |  |  |
| Flavodiet score | Ref. | 0.93 (0.88, 0.99) | 0.95 (0.89, 1.01) | 0.86 (0.81, 0.93) | 0.83 (0.77, 0.89) | <0.001 |
| Total flavonoids | Ref. | 0.95 (0.89, 1.01) | 0.94 (0.88, 1.00) | 0.89 (0.83, 0.96) | 0.85 (0.79, 0.92) | <0.001 |
| Impaired physical function (n events = 11241) | | |  |  |  |  |
| Flavodiet score | Ref. | 0.91 (0.86, 0.97) | 0.92 (0.87, 0.98) | 0.85 (0.80, 0.91) | 0.85 (0.80, 0.91) | <0.001 |
| Total flavonoids | Ref. | 0.97 (0.91, 1.03) | 0.93 (0.87, 0.99) | 0.89 (0.84, 0.95) | 0.89 (0.83, 0.95) | <0.001 |
| Poor mental health (n events = 5282) | |  |  |  |  |  |
| Flavodiet score | Ref. | 0.97 (0.90, 1.06) | 0.97 (0.89, 1.06) | 0.89 (0.82, 0.98) | 0.90 (0.82, 0.98) | 0.006 |
| Total flavonoids | Ref. | 0.89 (0.82, 0.97) | 0.93 (0.85, 1.02) | 0.92 (0.84, 1.00) | 0.89 (0.81, 0.98) | 0.114 |
| Hazard ratios (95% CI) for frailty, physical impairment and poor mental health obtained from Cox proportional hazards models adjusted for baseline age, questionnaire cycle, ethnicity, smoking status, marital status, menopausal status, family history of myocardial infarction, diabetes and cancer, multivitamin use, use of aspirin, use of other medications, history of hypertension, hypercholesterolemia, diabetes, myocardial infarction, and stroke, physical activity, BMI, and intakes of alcohol, total energy, meat, nuts, saturated fat, polyunsaturated fat, trans fat, cereal fibre, and soft drink (Model 2). | | | | | | |
